# Magnetically driven microfluidics for isolation of circulating tumor cells

**DOI:** 10.1002/cam4.3077

**Published:** 2020-04-23

**Authors:** Laan Luo, Yongqing He

**Affiliations:** ^1^ School of Chemical Engineering Kunming University of Science and Technology Kunming China; ^2^ Chongqing Key Laboratory of Micro‐Nano System and Intelligent Sensing Chongqing Technology and Business University Chongqing China

**Keywords:** circulating tumor cells, ferrofluids, magnetic field, microfluidics

## Abstract

Circulating tumor cells (CTCs) largely contribute to cancer metastasis and show potential prognostic significance in cancer isolation and detection. Miniaturization has progressed significantly in the last decade which in turn enabled the development of several microfluidic systems. The microfluidic systems offer a controlled microenvironment for studies of fundamental cell biology, resulting in the rapid development of microfluidic isolation of CTCs. Due to the inherent ability of magnets to provide forces at a distance, the technology of CTCs isolation based on the magnetophoresis mechanism has become a routine methodology. This historical review aims to introduce two principles of magnetic isolation and recent techniques, facilitating research in this field and providing alternatives for researchers in their study of magnetic isolation. Researchers intend to promote effective CTC isolation and analysis as well as active development of next‐generation cancer treatment. The first part of this review summarizes the primary principles based on positive and negative magnetophoretic isolation and describes the metrics for isolation performance. The second part presents a detailed overview of the factors that affect the performance of CTC magnetic isolation, including the magnetic field sources, functionalized magnetic nanoparticles, magnetic fluids, and magnetically driven microfluidic systems.

## Graphical abstract

This review aims to introduce two principles of magnetic isolation and recent techniques, facilitating research in this field and providing alternatives for researchers in their study of magnetic isolation. Researchers intend to promote effective CTC isolation and analysis as well as active development of next‐generation cancer treatment.

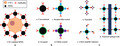

## 1 INTRODUCTION

Cancer is the second major cause of death in the modern world despite advances in therapy.^1^ Owing to chemotherapy, surgery, and development of targeted therapies, treatment of primary tumors has steadily progressed; however, metastasis remains frequent and leads to 90% of cancer deaths.^2^ Metastasis of cancer cells from primary tumors is a multistep process in which they detach and invade distant tissues via blood circulation.^3^ The cancer cells (a) infiltrate into the adjacent tissue [epithelial‐mesenchymal transition (EMT)], (b) migrate into the blood circulation of peripheral blood (intravasation), (c) escape immune system attacks and survive in circulation, (d) exit the bloodstream (extravasation), and (e) proliferate and develop a newly formed tumor in the distal organs [mesenchymal‐epithelial transition (MET)]. The cells that detach from the primary tumor and flow through the blood are called circulating tumor cells (CTCs).

CTCs were first discovered in 1869 during an autopsy of a patient with metastatic cancer by Thomas Ashworth^4^ who observed small numbers of cells in the blood of the patient, which resemble primary tumor cells. This observation implied that for the cancer cells to have reached the distant site, they would have had to be transported through the blood. CTCs have been demonstrated to be present in the blood and bone marrow of patients with lung,^5^, ^6^ liver,^7^ breast,^8^, ^9^, ^10^ prostate,^11^ and colon^12^ cancer. These cells have been shown to exist not only in patients with metastatic diseases but in those with apparently localized tumors.^13^ CTCs have clinical potential as prognostic biomarkers to predict treatment efficacy, progression‐free survival, and overall survival in patients.^14^, ^15^ CTCs that entered the circulation could be an excellent surrogate biomarker not only for prognosis but for disease detection and monitoring.^16^, ^17^, ^18^, ^19^ Prompted by their potential for application in cancer research and treatment, CTCs have drawn interest toward the development of strategies for improved isolation, enumeration, and characterization of CTCs.

One major limitation of CTC isolation and analysis is that CTC is extremely rare in the blood relative to blood cells, such as red blood cells (RBCs) and white blood cells (WBCs), presenting formidable technical and analytical challenges. Approximately 1 to 100 CTCs are found in 1 mL of peripheral blood from a cancer patient.^20^, ^21^, ^22^ Mature RBCs exhibit distinct physical, chemical, and biological properties that facilitate their removal from blood, and WBCs share numerous common properties with CTCs, resulting in high levels of WBC contamination in many isolation methods.^23^ Thus, techniques need to be developed to isolate these CTCs from blood, and important performance metrics for these methods include the high recovery rate and reasonable purity of CTCs, the ability to quickly process large volumes of blood (eg, throughput ∼7.5 mL/h), and maintain cell integrity.^24^

Microfluidic systems with their network of microchannels have been widely used for chemical, biological, and medical applications due to their ability to analyze or process fluids and suspensions with volumes in the sub‐microliter range.^25^, ^26^ In comparison with the traditional techniques, the miniaturization of microfluidic manipulations has the features such as small sample volume requirement, fast processing times, multiplexing capabilities, and large surface area‐to‐volume ratios.^27^, ^28^, ^29^ Based on these advantages, various microfluidic platforms have been developed for isolation CTCs. Passive and active isolation techniques are two methods of isolating CTCs in a microfluidic system. Passive methods based on size‐based filtration ^30^, ^31^, ^32^ typically suffer from the low purity of isolated CTCs and difficulty of collection. Meanwhile, active techniques exploit various external forces, such as optical, acoustic, electrical, and magnetic forces. Photophoresis can locate light to the level of a single cell, but the risk of cell damage is present because highly focused beams sometimes generate excessive heat.^33^ Acoustophoresis can only isolate cells by differences in cell size, density, and compressibility due to the difficulty of integrating the acoustic transducer into a microfluidic device and the difficulty of controlling submicron‐scale cells.^34^, ^35^ Electrophoresis requires local circuitry and large potentials, which can cause its dissolved ions and surface potentials to damage cells.^36^, ^37^ By contrast, magnetophoresis has several distinct advantages, such as low cost and reduced sample consumption; in addition, it has no heating problems and requires no expensive external systems as an aid.^38^, ^39^

Currently, CTC isolation methods based on magnetically driven microfluidics can be broadly categorized into labeled methods and label‐free methods. Two main methods of labeled magnetic isolation are typically used: positive and negative selection. When a magnetic field is applied, CTC can be actively isolated using functionalized magnetic nanoparticles (MNPs). The specific antigen coupled MNPs can react with specific surface proteins on CTCs to achieve positive CTC selection.^40^, ^41^, ^42^, ^43^ CTCs shed from primitive tumors are highly heterogeneous due to the diversity of cancer cells, including epithelial cancer cells such as gastric cancer, mesenchymal cancer cells such as osteosarcoma, and other cancer cells such as leukemia. This allows for a wide variety of antigens to label different CTCs,^44^ the most commonly used antigen is anti‐epithelial cell adhesion molecule (EpCAM). Alternatively, negative enrichment of CTC can be achieved based on WBC depletion using anti‐CD45 surface antigens because the antigens can be specifically expressed on the surface of WBCs.^45^, ^46^, ^47^ Owning to inter‐patient and intra‐patient heterogeneity in tumor biology, especially in the case of EMT, the identification of CTC‐specific markers becomes complicated.^48^ Meanwhile, label‐free magnetic isolation uses magnetic fluids such as paramagnetic salt solutions or ferrofluids as media to isolate CTCs based on their difference in size from those of hematological cells.

**Figure 1:**
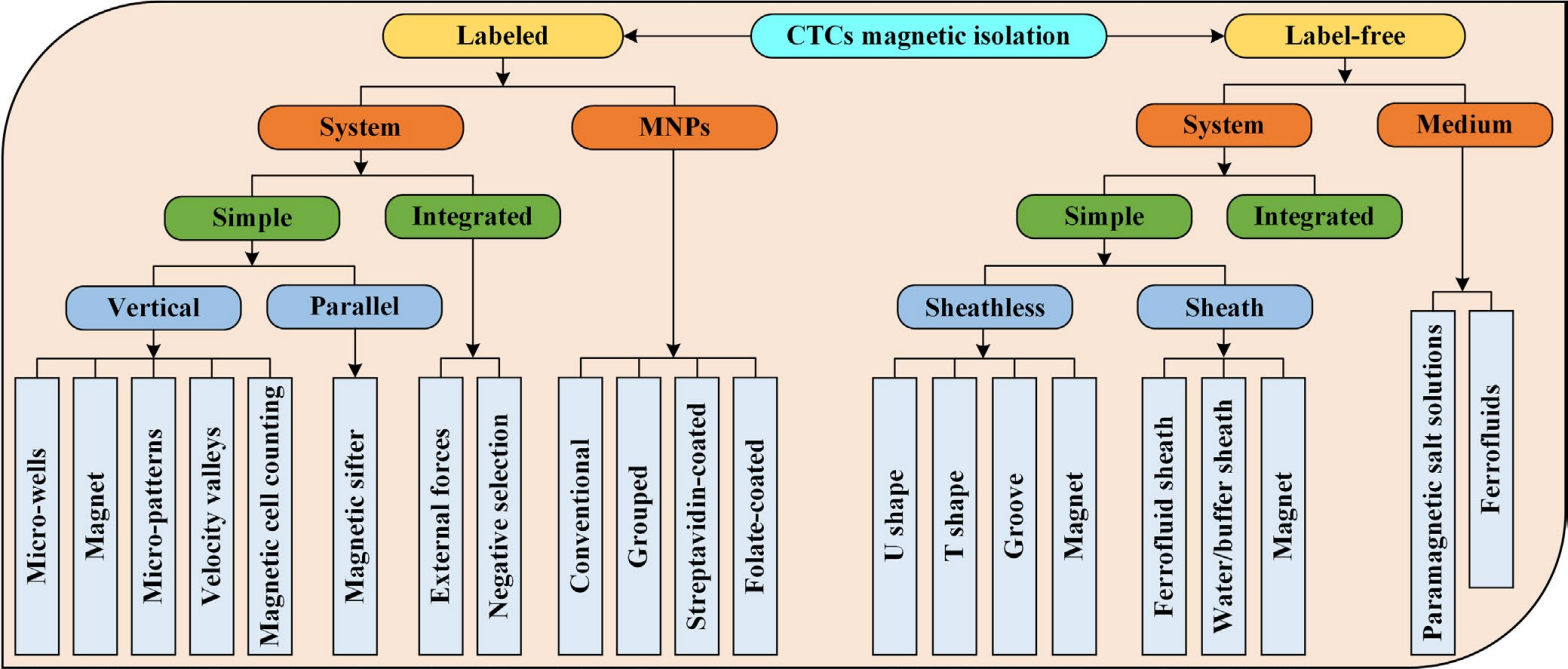
Schematic structure of this article

This article aims to review the fundamental principles of magnetophoresis and its recent applications in microfluidic isolation of CTCs. The remaining sections are structured as follows. Section 2 describes three magnetic field sources, including electromagnets, permanent magnets, and soft magnets, as well as the metrics for characterizing CTC isolation performance, including purity, recovery, and yield. Section 3 summarizes the functionalized MNPs commonly used to label CTCs, which include conventional, grouped, streptavidin (SA)‐coated, and folate (FA)‐coated MNPs, as shown in Figure 1. Microfluidic systems used in positive magnetophoresis, which include simple and integrated systems, are reviewed and compared. Simple systems include the following: magnetic sifters, microwells, magnets, micropatterns, velocity valleys, and magnetic cell counting systems based on the position of the magnetic field source. Integrated systems include various external field force integration and negative selection techniques. Section 4 presents the medium used for label‐free isolation, which includes paramagnetic salt solutions and ferrofluids, as shown in Figure 1. It summarizes the existing microfluidic systems of negative magnetophoresis, including sheathless and sheath technologies, and compares the systems based on positive magnetophoresis. Finally, an outlook for this research field is presented.

## 2 BASIC CONCEPTS

### 2.1 Sources of magnetic field

The most important part of the magnetically driven microfluidic device is the magnetic field source, and several magnetic field sources are currently employed to obtain the magnetic field and gradient.

The first choice is an electromagnet that is suitable for generating variable magnetic field gradients and uses different designs, such as single‐wire and microcoil arrays. This approach can dynamically reconfigure the magnetic field mode but has the disadvantage of producing Joule heating, which markedly limits the maximum of the generated magnetic field, thereby narrowing the field gradient and potentially exerting an adverse effect on cell viability.^49^

The second choice is the permanent magnet. Their high‐gradient micron‐sized permanent magnets can be integrated into the chip very close to the microchannel, which can achieve the magnetic force required for CTC isolation without any external power and involves a simple setup.^50^ Moreover, permanent magnets in several shapes and arrangements can be positioned near the microchannel to create the magnetic field gradients needed to isolate the CTCs.

A third method is to create a localized strong magnetic field gradient within the microfluidic device by embedding a soft ferromagnetic element at the bottom of the microchannel. This micropattern flux concentrator allows for a substantially stronger magnetic field than that of electromagnets while avoiding heat generation.

### 2.2 Positive and negative magnetophoresis

**Figure 2:**
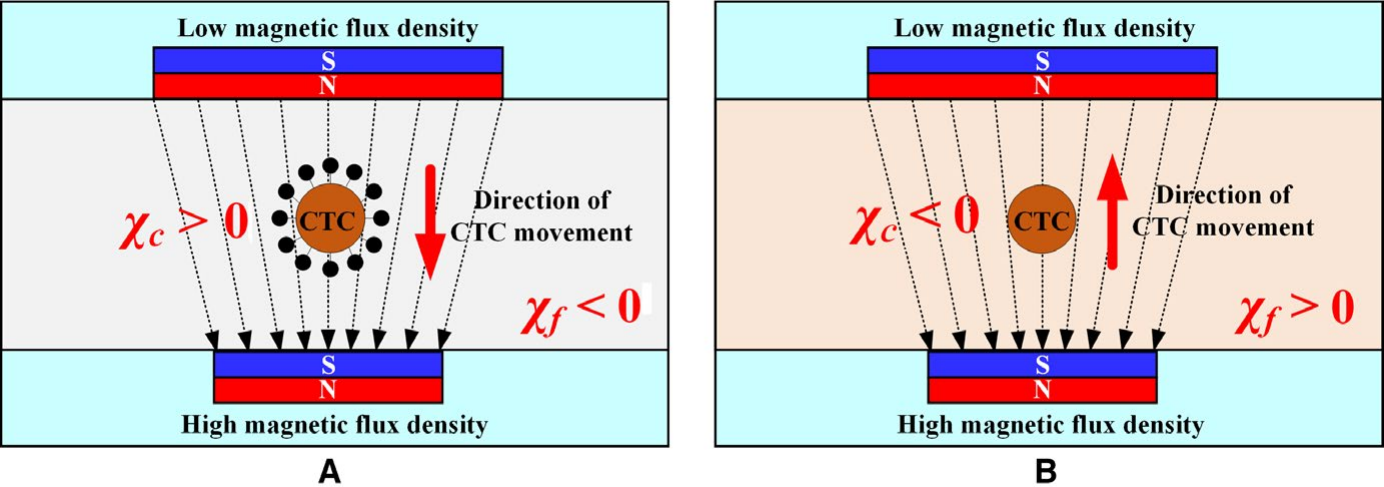
Mechanism of positive magnetophoresis and negative magnetophoresis.^120^ A, Positive magnetophoresis. *χ*_c_ is larger than *χ*_f_ in which case the CTCs can be drawn toward the maxima of the nonuniform magnetic field. B, Negative magnetophoresis. When diamagnetic CTCs (*χ*_c_ ≤ 0) are dispersed in a magnetic medium (*χ*_f_ > 0), the CTCs are drawn toward the minima of the nonuniform magnetic field

Isolation and sorting CTCs by deflection have been explored extensively. Two basic concepts for CTC isolation using magnetic fields have been identified: positive magnetophoresis (Figure 2A) and negative magnetophoresis (Figure 2B). Positive magnetophoresis means that the magnetic susceptibility of CTC is higher than that of medium, which requires marking CTC with MNPs, so that CTC is attracted to the region with higher magnetic field strength. In contrast, negative magnetophoresis uses a magnetic fluid with a higher susceptibility to repel CTCs toward areas with lower magnetic field strength. The detailed process is as follows: the unlabeled CTCs are placed in a uniform magnetic fluid to act as “magnetic holes^51^”; the magnetic field gradient generated by the permanent magnet will attract the magnetic medium to push the “magnetic holes (CTCs)” away; therefore, the CTCs can be continuously isolated in a label‐free manner.

The mobility of CTCs relative to environmental fluids depends on two factors: (a) the difference in volume magnetic susceptibility between the CTCs and the surrounding fluid and (b) the gradient of the magnetic field, as shown in Equation (1).^52^, ^53^

(1) $$F_{m}=\left[N_{\text{nano}}\left(\chi_{\text{nano}}-\chi_{f}\right)V_{\text{nano}}+\left(\chi_{c}-\chi_{f}\right)V_{c}\right]\frac{(B\cdot \nabla )B}{\mu_{0}},$$

where ***F*_m_** is the magnetic force acting on CTCs; *μ*_0_ is the magnetic permeability of the vacuum; ***B*** is the magnetic field strength; *N*_nano_ is the number of MNPs bound per CTC; *V*_nano_ is the volume of MNPs; *χ*_nano_, *χ*_f_, and *χ*_c_ are the volumetric magnetic susceptibility of the MNPs, surrounding fluid, and CTCs, respectively.

For negative magnetophoresis, the magnetic force can be written as^54^

(2) $$F_{m}=-V_{c}\mu_{0}\left(M\cdot \nabla \right)H,$$

where *V*_c_ is the volume of the individual cell; the effective magnetization of the ferrofluids around the cell ***M*** (could be determined by the classical Langevin theory) is collinear with a static magnetic field ***H*** produced by the permanent magnet.

### 2.3 Performance metrics

To achieve ideal CTCs isolation, high purity and high recovery rates are necessary while maintaining the viability and integrity of the CTCs for downstream characterization and molecular analysis. High‐throughput isolation, which refers to the sample volume or the number of CTCs handled within a given time,^21^ also needs to be conducted.

Purity is the ratio of CTCs isolated from the microfluidic system to the total number of isolated cells, as shown in Equation (3). Higher purity is advantageous for subsequent single‐cell analysis, but the purity may vary for different types and concentrations of CTCs and different means of microfluidic systems.

(3) $$\text{Purity}={\left(\frac{\text{Target cells}}{\text{Target cells}+\text{Background cells}}\right)}_{\text{output}}.$$

Recovery or isolation efficiency refers to the ratio of isolated CTC to total cells at the output of an isolation system, as shown in Equation (4). This parameter is closely related to purity because when the isolated CTC contains fewer other cells, the isolation efficiency is high.

(4) $$\text{Recovery}/\text{isolation efficiency}={\left(\frac{\text{Target cells}}{\text{Background cells}}\right)}_{\text{output}}.$$

Yield is the fraction of isolated CTCs relative to the number of CTCs in the original sample, as shown in Equation (5). Yield is important for CTC enumeration where it is essential to know the CTC concentration in the patient bloodstream.

(5) $$\text{Yield}=\frac{{\text{Target cells}}_{\text{output}}}{{\text{Target cells}}_{\text{input}}}.$$

## 3 LABELED ISOLATION OF CTCS

Labeled isolation of CTC refers to binding to a target antigen or a surface marker present on a CTC membrane by a specific antibody, which can then be isolated for further downstream analysis, such as DNA sequencing, to characterize the heterogeneity of CTCs.^55^ Anti‐EpCAM is the most widely used antigen. The labeled magnetically separated CTC uses functionalized MNPs, including conventional, grouped, SA‐coated, and FA‐coated MNPs coupled with anti‐EpCAM to form a stable CTC complex that is isolated in the presence of an external magnetic field. Moreover, various magnetically driven microfluidic systems have been designed to meet the high throughput and high efficiency of isolated CTCs. These systems include microwells, magnets, micropatterns, velocity valleys, and magnetic cell counting systems as well as integrated systems based on various external field forces or microchannel structures.

### 3.1 Types of functionalized MNPs

The distinct properties of MNPs, combined with general surface engineering techniques, have led to the emergence of magnetically isolated labeled cell methods that separate labeled cells from complex biological samples.^56^ The enhanced efficiency of these methods has a significant impact on both basic research and clinical applications. The MNPs coupled with the antibody are added to the cell system to be isolated, and the target cells are recognized, with the antibodies on the surface of the magnetic particles, to form stable complexes. The isolation of the identified cells from other cells is then affected by an external magnetic field. To maximize the bonding ability and isolation efficiency, and improve the sensitivity and efficiency of detection, MNPs with multiple functions have been reasonably designed.

MNPs usually consist of metal oxides (Fe_3_O_4_, γ‐Fe_2_O_3_, etc) or pure metals (Fe, Co, Ni, etc) because these materials exhibit high‐saturation magnetization. Pure metals possess good magnetic properties; however, their high toxicity and oxidative sensitivity render them unsuitable for biomedical applications without proper and stable surface treatment. Some studies^57^, ^58^, ^59^ used anti‐EpCAM‐modified γ‐Fe_2_O_3_ MNPs to isolate CTCs; however, many chose Fe_3_O_4_ MNPs because of their higher magnetic permeability than that of γ‐Fe_2_O_3_ MNPs.

The preparation of MNPs mainly includes physical,^60^ microbial,^61^ and chemical methods. Although physical methods can be prepared in batches, precise instruments are required. The microbial method is difficult to achieve large‐scale production due to the limitation of production rate and yield. In contrast, chemical methods can be used to prepare MNPs with different properties, which show great advantages in the synthesis, assembly, surface modification, and functional integration of MNPs. The chemical preparation methods^62^ include oxidation, chemical coprecipitation, hydrothermal, aerosol/vapor‐phase method, etc.

#### 3.1.1 Conventional MNPs

**Figure 3:**
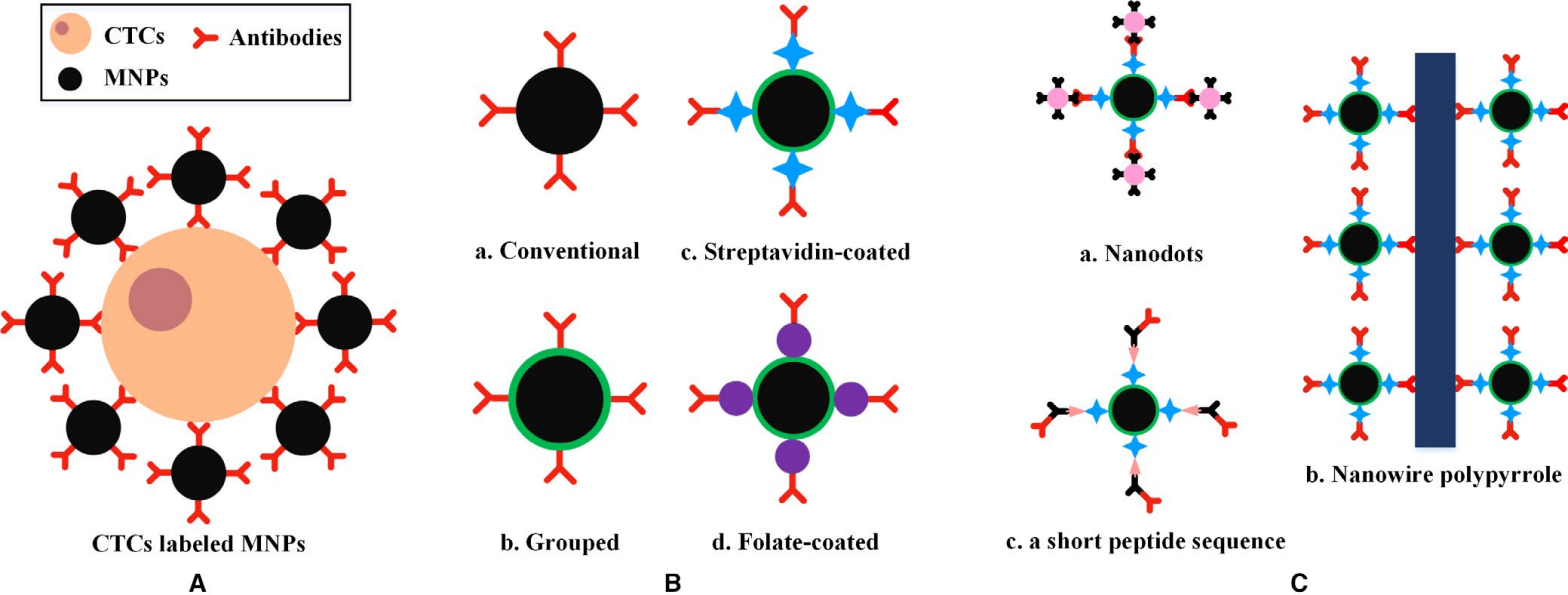
Functionalized MNPs. A, Schematic of CTCs combined with functionalized MNPs. B, Schematic of conventional, grouped, streptavidin‐coated, and folate‐coated MNPs. C, Three typical extended states of streptavidin‐coated MNPs, including nanodots, nanowire polypyrrole, and a short peptide sequence

Functionalized MNPs based on surface processing methods can generally be categorized into conventional, grouped, SA‐coated, and FA‐coated MNPs, as shown in Figure 3A,B. The method of directly coupling the antibodies to the surface of MNPs through a covalent bond is called a conventional method. The main considerations are the magnitude of the magnetic field and the structure of the microfluidic system for conventional MNPs. After being magnetized, the ferromagnetic micromagnets in the microfluidic system generate localized magnetic fields up to eightfold stronger than that without the micromagnets, thus enhancing the interaction between the CTC and the magnetic field.^63^ The use of high‐force magnetic ratcheting over arrays of magnetically soft micropillars with gradient spacing can purify the magnetic particle population and isolate cells based on the number of bound particles.^64^ A magnetic sifter in microfluidic chips can isolate CTCs with high‐throughput and distinguish heterogeneous cell populations.^65^ Positive selection methods, such as the three aforementioned approaches, can efficiently isolate cancer cells; however, they may undergo EMT and downregulate the expression of EpCAM. The use of a negative selection method for these cancer cells is more appropriate to effectively remove leukocytes by applying an external magnetic force, leaving an enriched target cell population (CTCs).^66^, ^67^ Moreover, a computational method was proposed to analyze the behavior of blood flow and evaluate the isolation efficacy using multiple design parameters, including the channel design, channel operational orientations (inverted and upright), and flow rates.^68^ A detailed description of the CTCs used in various magnetic isolation methods and their associated cancer cell lines is listed in Table 1.

**Table 1:** Summary of various functionalized MNPs

MNPs	Cell lines	Cancer types	Magnet	References
Conventional	MCF7 & SKBR3/COLO205/PC3	Breast/colorectal/prostate	Ni & NdFeB (N42)	[63]
	PC3 & LNCaP	Prostate	Permalloy & Neodymium (N52)	[64]
	H1650 & H1975	Non‐small cell lung	Magnetic sifter	[65]
	HCT8/Jurkat T	Colorectal/leukemia	Permanent	[66]
	A549	Lung	Permanent	[67]
	COLO205	Colorectal	NdFeB(N42)	[68]
Grouped	CL15	Lung	Gold & Neodymium	[69]
	MCF7 & MDA‐MB‐231	Breast	Permanent	[55]
	SKBR3/CAL120	Breast/mesenchymal	Neodymium	[70]
	TCHU147	Leukemia	BS0090	[71]
	MCF7	Breast	Permanent	[72]
	MCF7	Breast	Permanent	[73]
	MCF7/HepG2/HCT116/Jurkat T	Breast/hepatocellular/colorectal/leukemia	Permanent	[74]
SA‐coated	SKBR3/H460	Breast/lung	Permanent	[75]
	MCF7/HCT116/PaCa2	Breast/colorectal/pancreatic	Permanent	[76]
	SKBR3/HeLa	Breast/cervix	Permanent	[77]
	HCT116	Colorectal	Permanent	[78]
FA‐coated	ZR‐75‐1/HeLa	Breast/cervix	Neodymium	[79]
	A549&H1299&H157&H460&SPC‐A‐1/MCF7/A2780/Jurkat T	Non‐small cell lung/breast/ovarian/leukemia	Permanent	[80]
	MCF7/HCT116	Breast/colorectal	Permanent	[81]

Note

“Permanent” in the table indicates that a magnet is used in the original paper, but the specific model is not mentioned.

#### 3.1.2 Grouped MNPs

Microfluidic devices for CTC isolation provide effectively separate target cells with minimal nonspecific binding owing to shear forces generated by the fluid flow.^69^ However, the interaction between CTCs and surface antibodies is often insufficient because of the presence of laminar flow in the channels. Grouped MNPs can be well coupled to the antibody by reacting with a specific polymer and then immobilizing the CTCs in a specific solution to form a stable complex. The gold nanoslit surface plasmon resonance platform developed by Mousavi et al^69^ can efficiently capture and detect CTCs in human blood. Target cells are first captured with aminated MNPs and then in a microfluidic chip integrated with a gold nanoslit film for further analysis. Kwak et al^55^ synthesized hydroxylated MNPs using the hydrothermal method to isolate CTCs in a magnetic gradient‐based microfluidic chip and simultaneously characterized the state of CTCs with respect to EpCAM expression.

Carboxylated MNPs can be activated by 1‐ethyl‐3‐(3‐dimethylaminopropyl) carbodiimide or N‐hydroxysuccinimide (NHS) to form esters, which can easily immobilize antibodies or nanodots, providing a foundation for the isolation, detection, and analysis of CTCs. Nanodots, including polymer dots, quantum dots, carbon dots, and gold clusters, among others, can be used for bright imaging of biological imaging because of their superior photostability and biocompatibility with cells and tissues. Magnetic nanoprobes coated with polymer dots were synthesized by Pramanik et al.^70^ These nanoprobes can be used for targeted capturing and multicolor fluorescence mapping of heterogeneous CTCs and can distinguish targeted CTCs from nontargeted cells. Li et al^71^ developed DNA‐templated magnetic nanoparticle‐quantum dot‐aptamer copolymers that facilitate the isolation and counting of CTCs with high sensitivity and accuracy within 20 minutes. Mei et al^72^ incubated two antibodies (a tag‐DNA‐modified CK‐19 antibody and an MNP‐conjugated EpCAM antibody) together with CTCs, and further enriched the CTCs by magnetic isolation, thereby detecting single tumor cells in a 5‐mL blood sample. This method exhibits high sensitivity and provides convenience, that is, without the need for tumor gene extraction. Owing to the hydrophobicity of silane or the porosity of silica, they can be covered outside of MNPs and filled with other substances in the mesopores to enhance fluorescence for further bioimaging and analysis. Xu et al^73^ proposed a bionic TiO_2_ inverse opal photonic crystal. This structure can not only achieve high‐efficiency capture of CTCs by the combination of Fe_3_O_4_@C6@silane MNPs but can also enhance the fluorescence signal, facilitating real‐time monitoring. Fe_3_O_4_@C6@silane MNPs denote that Fe_3_O_4_ MNPs serve as core and encapsulate with silane. Coumarin 6 (C6) hydrophobic organic molecules are loaded into the interspaces between them, serving as imaging agent. Chang et al^74^ designed magnetic mesoporous silica nanoparticles into spherical and rod‐like morphologies. Although different shapes of MNPs achieved efficient enrichment of CTCs and fluorescence‐based detection, the performance of rod‐like MNPs was superior to that of spherical MNPs.

#### 3.1.3 Streptavidin‐coated MNPs

A stable amide bond can be formed by reacting an amino group on SA with an ester obtained from an activated carboxyl group on the surface of MNPs. Biotinylated antibodies can rapidly bind to MNPs because of the high affinity of the biotin‐streptavidin system. The combination of SA‐coated MNPs with other substances, such as nanodots,^75^ nanowire polypyrrole,^76^ or a short peptide sequence,^77^ can be used for the efficient capture and simple quantification of CTCs to diagnose and monitor cancer, as shown in Figure 3C. Moreover, owing to the good biocompatibility and surface modification of MnO_2_, as well as its quick dissolution by considerably low concentrations of oxalic acid at room temperature, SA‐coated Fe_3_O_4_@MnO_2_ MNPs can capture and release CTCs with good viability.^78^

#### 3.1.4 Folate‐coated MNPs

The choice of cell surface tumor‐specific antigens is key to increasing CTC isolation and detection rates because EMT during metastasis tends to result in increased loss of epithelial CTCs. The folate receptor is significantly overexpressed in various types of cancer, including breast, lung, kidney, ovary, colon, brain, and leukemia. The sensitivity and specificity of the isolation and detection of HeLa cells^79^ or non‐small cell lung cancer cells^80^ were enhanced by labeling folate‐coupled MNPs. Zhu et al^81^ coated folic acid and MNPs on the surface of RBCs and were then quickly adhered to CTCs to obtain CTC‐RBC conjugates. After treatment with RBC Lysis Buffer and centrifugation, CTCs were released and captured. This approach provides a method to efficiently capture ultralow‐density cells and achieve high purity. Moreover, numerous types of surface markers, including transferrin receptor,^82^ epidermal growth factor receptor,^83^ Fc‐mannose‐binding lectin,^84^ and mesenchymal N‐cadherin,^85^ have been proposed to reduce the loss of CTCs.

The effective dissociation of isolated CTCs from functionalized MNPs to facilitate the subsequent analysis and cultivation of CTCs is an important issue that needs to be solved urgently. Although the intelligent sensing interface based on nucleic acid aptamer^86^ and temperature‐sensitive materials^87^ has successfully achieved the reversible isolate and release of CTCs, the types of degradable magnetic nanomaterials are still relatively small, and the application effects need to be further improved.

### 3.2 Types of microfluidic systems

Low recovery and low purity may affect the ability to select primary treatment for metastatic disease and monitor the effectiveness of postoperative treatment and relapse.^88^ Low purity increases the risk of discriminating and distinguishing between CTCs and enriched cells. Poor detection of CTCs hampers the clinical standardization of conventional cancer prognosis. Isolation performance needs to be improved, loss and purity have to be reduced, and analysis time needs to be shortened. Microfluidic technology provides a powerful platform for the isolation and analysis of CTCs by handling small samples with high precision and integration.^89^

#### 3.2.1 Simple microfluidic systems

On the basis of whether the magnetization direction of the magnetic field is consistent with the direction of the fluid flow, simple microfluidic systems can be classified into parallel and vertical systems. CTC isolation based on the parallel principle usually involves a magnetic sifter. Meanwhile, vertical systems are further categorized into the following: microwells, magnets, micropatterns, velocity valleys, and magnetic cell counting systems. The types of CTCs and MNPs, volume flow rate (*Q*), and isolation efficiency (*η*) in various simple microfluidic systems are listed in Table 2.

**Table 2:** Summary of simple microfluidic systems

System	Cell lines	Cancer types	MNPs	*Q *(mL/h)	*η* (%)	Clinical validation	References
Magnetic sifter	H1650	Non‐small cell lung	SA‐coated	10	91.4	Yes	[90]
	MCF7/HCC827	Breast/non‐small cell lung	Conventional	—	—	Yes	[91]
Microwells	THP1	Leukemia	Grouped	3	62	No	[92]
	M6C	Mouse breast	SA‐coated	1.2	~90	No	[93]
	HCT116	Colorectal	SA‐coated	0.54	92	No	[94]
	Hep3B	Hepatocellular carcinoma	SA‐coated	1.5	~90	No	[95]
Magnet	COlO205/SKBR3	Colorectal/breast	Conventional	10	90	No	[96]
	SKBR3/PC3/COlO205	Breast/prostate/colorectal	Conventional	2.5	>90	Yes	[97]
Micropatterns	SKBR3	Breast	Conventional	5	~90	Yes	[88]
	MCF7	Breast	Conventional	2.4	~93	No	[98]
	COlO205	Colorectal	Conventional	2.5	69.1	No	[99]
	MCF7	Breast	Conventional	0.02	90.8	No	[100]
	COlO205	Colorectal	Conventional	2.5	95.6	No	[101]
	RAW 264.7	Mouse leukemic macrophage	SA‐coated	—	>90	No	[102]
	SUM149	Breast	SA‐coated	3	—	No	[89]

**Figure 4:**
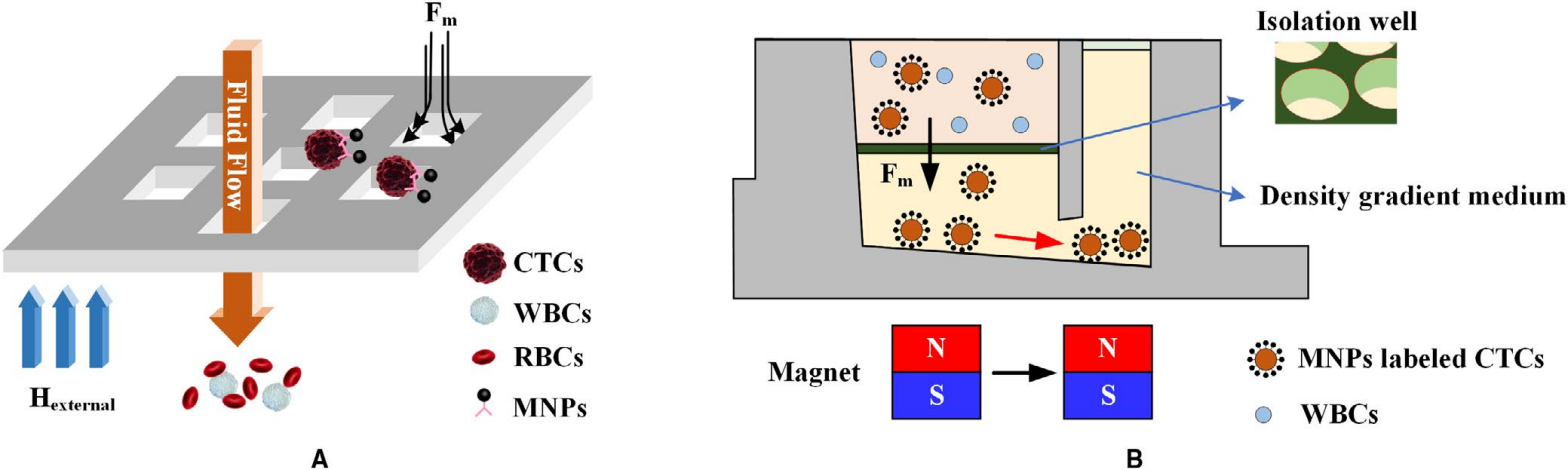
The magnetic sifter. A, Isolation principle. A whole blood sample containing MNP‐labeled CTCs is pumped through the pores. The CTCs are captured at the edge of the pores where a high magnetic field gradient exists, while unlabeled cells pass through the pores.^90^ B, Isolation of CTCs in density gradient media. The blood sample is loaded onto a density gradient medium. The CTCs are separated by a magnetic field gradient and then collected by moving the magnet from the separation chamber to the collection chamber^91^

The magnetic sifter^65^, ^90^ uses a flow‐through fluidic array structure that produces a large equivalent magnetic force at each pore and a uniform rinse flow for cell isolation, as shown in Figure 4A. Labeled CTCs are subjected to a large magnetic trapping force toward the edge of the pore, and normal blood cells smoothly pass through the magnetic pores. The captured CTCs can be imaged directly on the magnetic sifter array. They can then be released and collected by removing the external magnetic field and rinsing through the device with water or buffer solution. A density gradient medium can be added to the bottom of the magnetic sifter (Figure 4B), allowing the efficient separation and purification of CTCs by a vertical magnetic force in modified well plates.^91^

**Figure 5:**
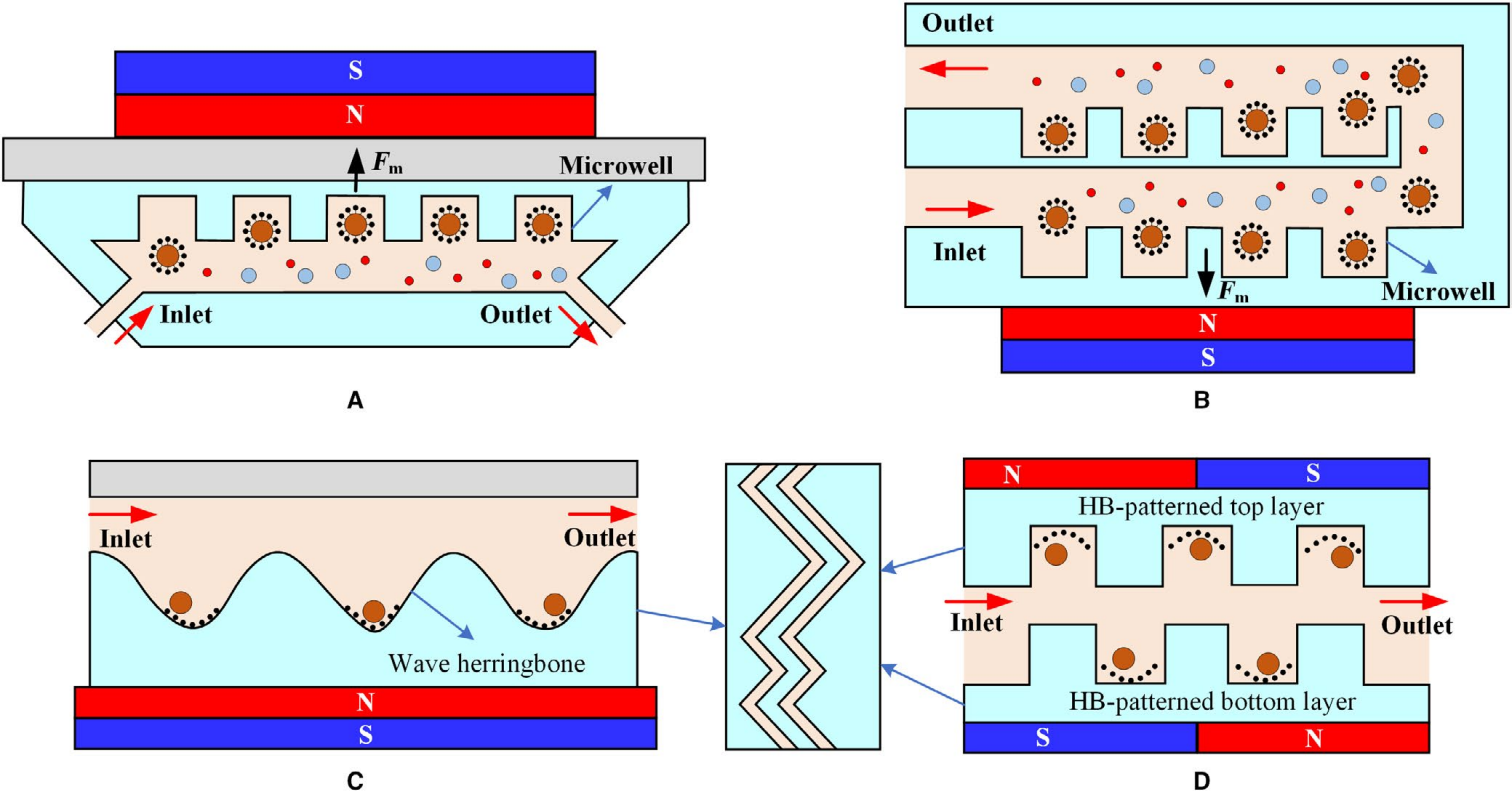
The microfluidic microwell device. A, Cell sedimentation caused by gravity can be reduced by placing the magnet directly above the microfluidic system.^92^ B, CTCs can be captured twice with the “double collection” structure to effectively prevent them from escaping.^93^ C, The vortex effect produced by the herringbone structure increases the chance of CTCs colliding with the wall.^94^ D, The double‐layer HB structure improves the performance of the device^95^

The direction of the pore in the magnetic sifter is consistent with the magnetization direction of the magnet. The same is true for microwells, where magnets are placed on the same centerline of the microwell (Figure 5). The magnetic field along the device increases in uniformity by adding the microwells. The labeled CTCs can remain in the microwell array while the uncaptured blood cells are washed away after high‐speed washing. In Figure 5A, a magnet placed on top of the microfluidic device prevents cell sedimentation caused by gravity.^92^ Figure 5B shows the “double collection” of the microfluidic structure. After the first capture, the CTCs that escape are captured in the second microwells, improving the capture efficiency.^93^

Owing to the fragility of CTCs, the sharp groove pattern may physically damage the cells during collision. This sharp groove pattern can be smoothed into a wavy pattern, that is, a herringbone (HB) structure similar to that in Figure 5C to induce a vortex effect. This effect can create passive turbulence and increase the probability of CTCs colliding onto the device wall.^94^ The internal regions become expanded because of the inherent HB structure. The HB groove as a semi‐closed space can locally capture the CTCs when a magnetic field is applied. Figure 5D presents the design of the HB pattern on both the top and bottom layers of the microchip, which improves the performance of the device.^95^

**Figure 6:**
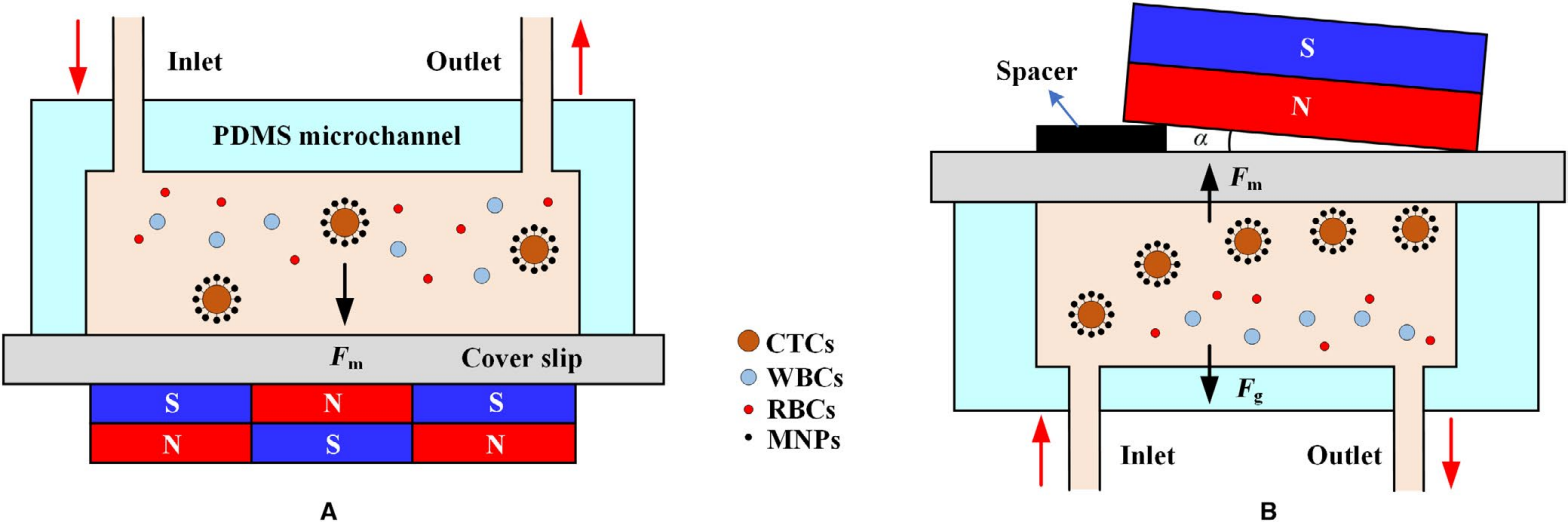
Arrangement of magnets. A, Alternately arranged magnets with opposite polarities form a large gradient.^96^ B, The insertion of a spacer provides a large magnetic field strength at the back end of the microchannel to reduce the loss of CTCs^97^

Both the magnetic sifter and the microwells are based on the adjustment of the internal structure of the microfluidic system. The external magnetic field source directly changes the magnetic field strength acting on the CTCs. The magnetic field gradient effectively attracts MNPs that essentially act as small dipoles. The sharp magnetic field gradient near the array magnets with alternating polarities combines with the thin, flat dimensions of the microchannel, resulting in efficient capture of labeled CTCs,^96^ as shown in Figure 6A. Inserting a spacer between the microchannel and the permanent magnet, that is, a tilted permanent magnet (Figure 6B), not only reduces the magnetic force at the entrance to eliminate cell aggregation but enhances the magnetic field at the exit of the microchannel to capture all CTCs that may escape in the weaker magnetic field.^97^

**Figure 7:**
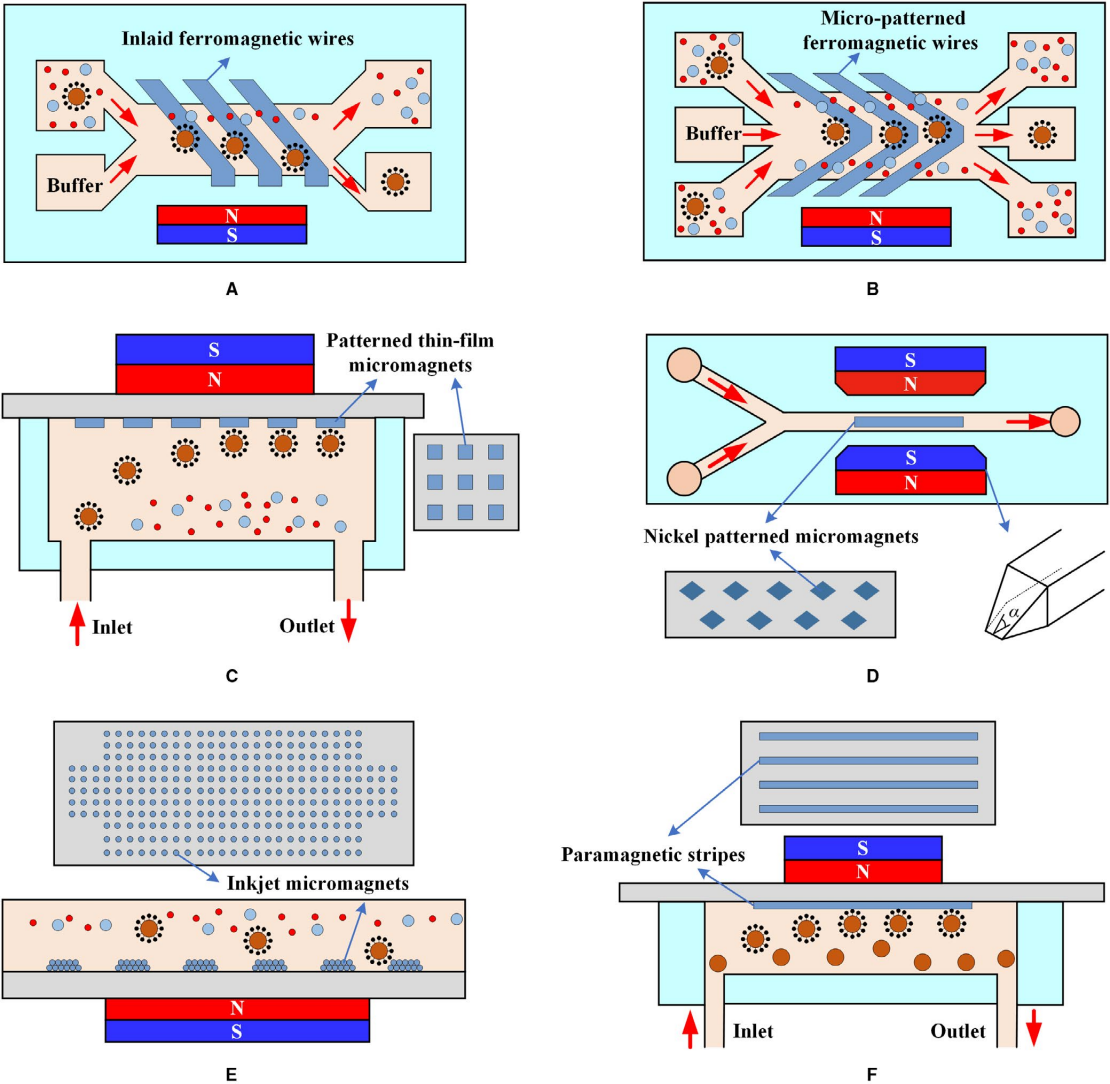
Arrangement of micropatterns. A, Isolation is performed via lateral magnetophoresis induced by a high‐gradient magnetic separator, a ferromagnetic wire array inlaid in the bottom substrate of a microchannel.^88^ B, The principle of CTC separation by lateral magnetophoresis is based on a V‐shaped ferromagnetic wire array in the microfluidic system.^98^ C, When the blood sample flows through the microchannel, CTCs are attracted to permanent magnets, which are placed outside the channel and trapped by thin‐film micromagnets on the channel substrate, whereas normal blood cells are unaffected and flow out of the channel.^99^ D, The trapezoidal solenoid produces a focused magnetic field that captures CTCs on micromagnets at the center of the channel.^100^ E, Inkjet printing can deposit a defined magnetic pattern on any substrate to facilitate CTC isolation.^101^ F, The paramagnetic strips produced by molding enhance the efficiency of isolation^102^

The magnetic field strength decays rapidly with distance. Thus, additional magnetic structures (micropatterns) should be placed within the microfluidic channel to provide a robust means of creating a consistent field distribution in a microfluidic device. Nickel as a soft magnetic material can magnetize and demagnetize with the application or removal of the external magnetic field. Nickel micropatterns are often used to render microfluidic systems reusable. Figure 7 shows the arrangement of various micropatterns. These micromagnets operate using the same principle: after being magnetized by an external magnetic field, micromagnets generate a local strong magnetic field, enhancing the attractive interaction between CTCs and microchannels.

Lateral magnetophoresis generated by a ferromagnetic wire array embedded in the bottom of a microchannel is one of the common means of enhancing the local magnetic field, as shown in Figure 7A,B. At a small distance near the sidewall, the ferromagnetic wire is almost perpendicular to the sidewall of the channel to form a corner (Figure 7A) where aggregation and stacking of CTCs are avoided.^88^ The principle of lateral magnetophoresis also applies for the V‐shaped nickel‐cobalt soft magnetic wire array described by Park et al,^98^ as shown in Figure 7B. The micromagnets are designed using an alternating pattern to create strong local magnetic field gradients during magnetization and multiple distributed capture sites (Figure 7C). This process illustrates a multidimensional approach involving permanent magnets for long‐range attraction, as well as thin‐film micromagnets for short‐range retention.^99^ Jaiswal et al^100^ used a C‐shaped solenoid to generate an external magnetic field that subsequently magnetizes nickel micromagnets. The trapezoidal geometry of the solenoid arm was designed to produce a focused magnetic field that causes the CTCs to be pulled toward the micromagnets, as shown in Figure 7D. Moreover, a noncontact, layer‐by‐layer, maskless inkjet printing technique was reported by Chen et al^101^ in which MNPs are used as printing ink to produce micromagnets, as shown in Figure 7E. Sun et al^89^, ^102^ used cobalt ferrite nanoparticles as raw materials in the production of paramagnetic strips (Figure 7F) by molding, resulting in enhancement of up to fourfolds in CTC capture. Two‐stage magnetic isolation in continuous flow can effectively improve the purity. Lin et al^103^ fabricated a flyover‐style microfluidic chip to achieve high‐purity WBCs isolation. Magnetic bead‐labeled WBCs are first sorted laterally in the channel with a micro‐nickel structure, and then they were continuously flowed into a flyover‐style channel for vertical isolation. This two‐stage isolation method enables the purity of WBCs to reach 93.2%.

Micropatterns consisting of ferromagnetic wires or micromagnets retain the high sensitivity of magnetic isolation and the high specificity of immunological recognition. The micropatterns can also be easily adapted to different targets, such as different CTC types or different CTC stages, providing a promising platform for driving clinical and transformational applications.

The development of high‐performance microfluidic systems for CTC isolation and analysis, such as magnetic sifters, microwells, and micropatterns, as well as allowing for more sensitive CTC isolation and measurements, is a rapidly evolving field. However, individual tumors are highly heterogeneous and contain numerous subpopulations of cells, hence the possible heterogeneity of the CTCs of individual patients, including subpopulations that are correlated at different degrees with the development of metastatic disease.^104^ The aforementioned device only attempts to isolate the collection of all CTCs in the sample, which may underestimate the number of CTCs and miss a critical subpopulation. Monitoring the distribution of CTC populations and classifying them based on surface marker expression are necessary to elucidate the clinical relevance of blood CTCs, rather than merely isolating and counting CTCs.

**Figure 8:**
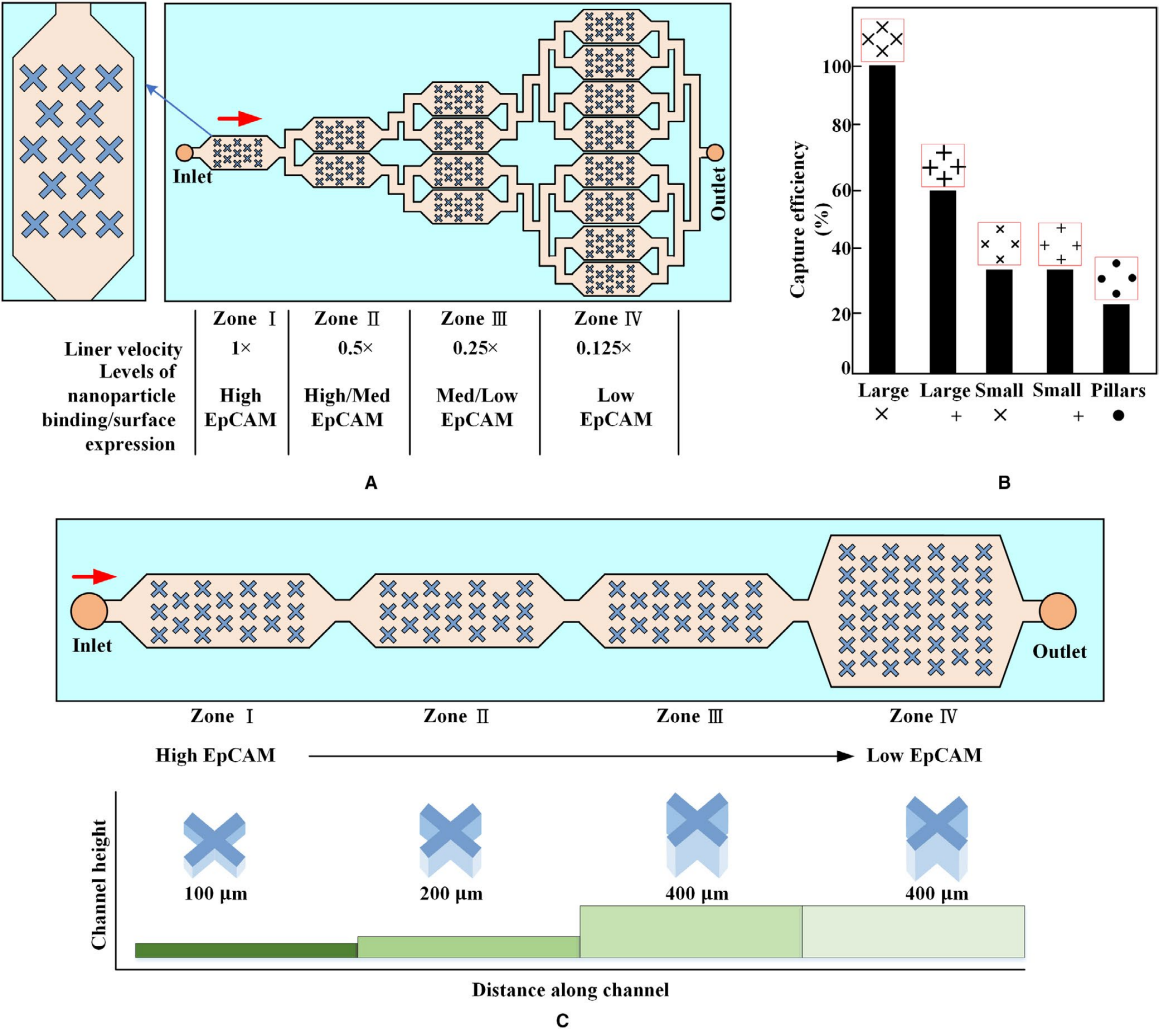
Velocity valley. The chip is sandwiched between arrays of permanent magnets with alternating polarities. A, Highly labeled CTCs exhibit high magnetic susceptibility to being captured in zones in which the drag force is higher, whereas poorly labeled cells continue moving and only become captured once they enter a zone with low linear velocity.^105^ B, Effect of trap geometry on capture efficiency of CTCs. Large X‐shaped structures are most efficient at capturing cells.^105^ C, Schematic of the cell sorting device with four zones of decreasing average linear velocity. Cells with high levels of surface markers are captured in the first zone, whereas cells with low levels of surface markers are captured in the final zone^107^

Kelley et al from the University of Toronto reported on a technique called velocity valley for CTC spatial sorting and profiling,^105^, ^106^ which isolates CTCs with different phenotypes into discrete spatial bins, as shown in Figure 8. The microfabricated structure within the fluidic device creates a localized pocket of low‐flow velocity; thus, regions that strongly favor the accumulation of targeted cells are created. These structures in fluidic zones with varying volumes can change the linear velocity of the flowing solution. Figure 8A presents a velocity valley chip containing four sorting zones. CTCs with a large nanoparticle population are captured by the first compartment within the chip due to their high linear velocity. The velocities of the following three regions are gradually decreased by a factor of two. The linear velocities of the different compartments depend on the range of expression levels of EpCAM in the CTCs. The X‐shaped structure is optimal for efficient CTC isolation, as shown in Figure 8B. The drag force in each sequential zone decreases by a factor of two via increasing the microchannel cross section (Figure 8C). The device can reduce nonspecific cell adhesion fivefold and isolate CTCs with a 100‐fold range of surface marker expression.^107^

**Figure 9:**
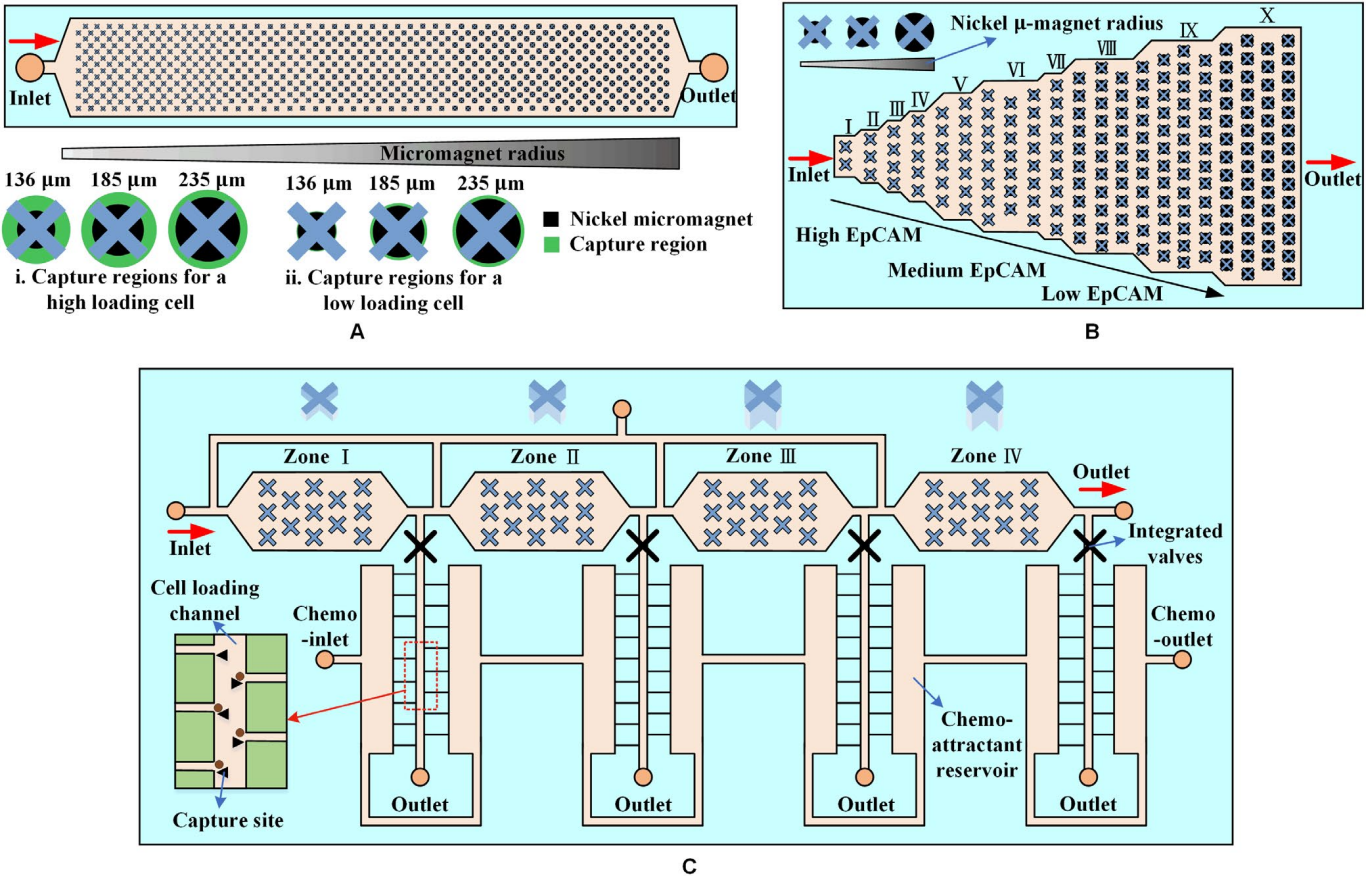
Magnetic cell counting system. A, An array of X‐shaped structures generates low‐flow regions; round nickel micromagnets are patterned within the channel to enhance the external magnetic field, and the micromagnets increase in size along the length of the channel. Arrays of magnets applied to the top and bottom of a microfluidic chip generate an external magnetic field.^108^ B, A device with 10 capture zones. The design reduces fabrication time and costs more than threefold. This device facilitates downstream analysis of CTCs by minimizing the chip‐scanning time by fluorescence microscopy and subsequent image processing.^110^ C, Cells are first sorted according to the levels of a surface marker, such as EpCAM. High‐ and low‐EpCAM cells are captured in Z1 and Z4, respectively. After EpCAM sorting, cell subpopulations extracted from each zone are subjected to chemotactic phenotype sorting^111^

Kelley et al combined the velocity valley with micropatterns to develop a magnetic cell counting system (MagRC),^108^, ^109^ as shown in Figure 9. The nickel micromagnets are concentrically positioned within the X‐shaped microstructure to create low‐flow regions and high magnetic field gradients, which is ideal for capturing low magnetic level labeled CTCs (Figure 9A). The local magnetic force within the device is designed to vary systematically via the micromagnet array (Figure 9A). MagRC also can simultaneously capture and sort CTCs expressed by different surface markers with high sensitivity and high efficiency. They^110^ increased the width of the capture zones steadily to shorten the length of the device by half, which not only maintains high capture efficiency but improves manufacturing yield, as shown in Figure 9B. This device allows CTC phenotypes to be profiled with sufficient resolution, particularly when the number of CTCs is considerably low. They also presented another device based on MagRC to profile the behavior of heterogeneous cell subpopulations along two independent phenotypic axes,^111^ as shown in Figure 9C. The labeled CTCs were first sorted based on differences in the expression of surface markers. Subsequently, these subsets were isolated into subpopulations corresponding to migration profiles generated in response to a chemotactic agent.

#### 3.2.2 Integrated microfluidic systems

Various microfluidic systems have been developed, including magnetic sifters, microwells, micropatterns, velocity valleys, and MagRCs to isolate CTCs from extracted blood samples; however, several challenges persist because of the low abundance, morphology, and heterogeneity of CTCs. Accordingly, an advanced CTC isolation and analysis technique that combines high throughput, purity, integrity, automation, and compatibility with established workflows should be developed. Integrated microfluidic systems have emerged, combining magnetic isolation with other forces from electrokinetics, acoustics, or optics.

**Figure 10:**
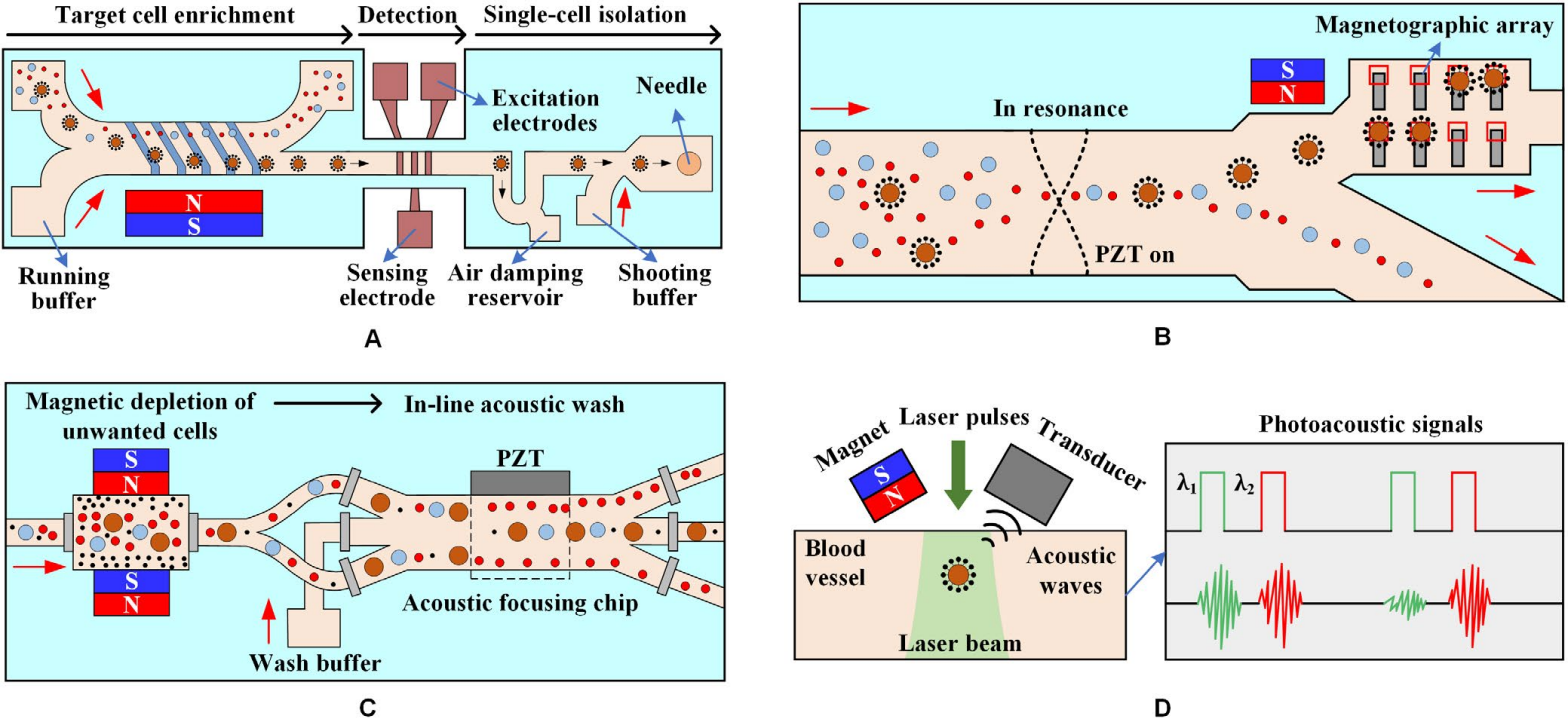
The microfluidic system combines several external forces. A, The lateral magnetophoretic microseparator is fabricated using a bottom glass substrate with an inlaid ferromagnetic Permalloy wire array positioned at an angle of 5.7° to the direction of the flow. The microdispenser, including the impedance cytometer and the microshooter, is developed using a glass substrate with patterned gold electrodes.^112^ B, Cells are acoustically focused to the center of the microchannel. Magnetically labeled cells are then deflected to the nonresonant portion of the microchannel via a gradient magnetic field. Finally, an array of micromagnets locally attracts magnetically labeled cells into microwells for on‐chip staining and analysis.^113^ C, The blood sample passes through magnetic depletion that removes > 98% of unwanted blood cells, followed by an in‐line acoustic focusing and washing step, which removes debris and concentrates the sample prior to cell sorting.^114^ D, CTCs targeted by two‐color nanoparticles can be illuminated by laser pulses at wavelengths of 639 and 900 nm with a delay of 10 μs. The laser beam is delivered either close to the external magnet or through a hole in the magnet by a fiber‐based delivery system^115^

Single‐cell separation has also been proposed,^112^ which involves the use of a lateral magnetophoretic microseparator, an electrical impedance cytometer, and a single‐cell microshooter, as shown in Figure 10A. The CTCs were first enriched using the lateral magnetophoretic microseparator. Since CTCs are generally larger than normal blood cells, the sizes of the enriched CTCs were then electrically identified using the impedance cytometer by sensing of amplitude modulation. Finally, the single‐cell microshooter would transfer the CTCs into single wells of standard containers individually.

Shields et al^113^ proposed a microfluidic platform consisting of three modules, as shown in Figure 10B. The cell mixture was first rapidly focused to the acoustic stagnation point in the presence of acoustic standing waves. The labeled CTCs were then isolated from normal blood cells in a magnetic field gradient. A periodic array of microwells with underlying micromagnets was designed in the last module to capture individual CTCs for on‐chip staining and analysis. Alternatively, the leukocytes and WBCs are first depleted magnetically, and the cells are washed and focused by acoustic to pre‐enriched CTCs prior to cell sorting.^114^ This integrated microfluidic system is shown in Figure 10C.

Galanzha et al^115^ have developed a platform (Figure 10D) for in vivo magnetic enrichment and detection of CTCs in combination with two‐color photoacoustic flow cytometry. Gold‐plated carbon nanotubes coupled with FA were used as a second contrast agent for photoacoustic imaging to enhance detection sensitivity and specificity. This platform integrates in vivo multiple targeting, magnetic enrichment, signal amplification, and multicolor recognition, allowing the concentration of CTCs from a large volume of blood in the vessels.

As an alternative to “positive selection,” “negative selection”^114^ of labeled blood cells, such as leukocytes, reduces the contamination of CTCs and increases their viability; however, the approach to labeling a large number of WBCs in whole blood is worth considering.

**Figure 11:**
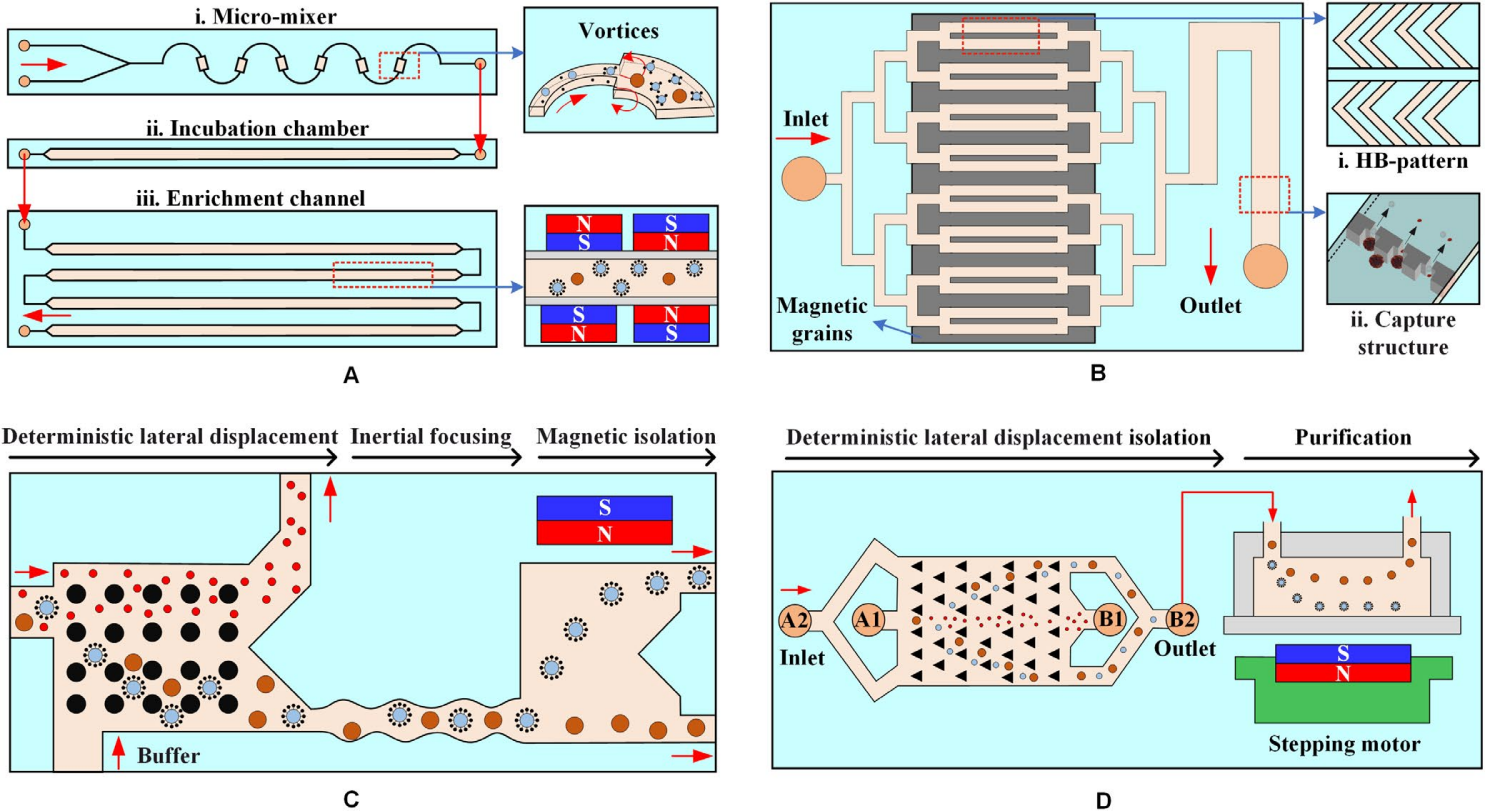
The microfluidic system based on negative selection method. A, The μ‐MixMACS chip has three parts: a microfluidic mixer to enhance the interaction between CD45‐conjugated MNPs and WBCs, an incubation chamber for stable MNP conjugation to the WBCs, and a magnetic activated cell sorter (MACS) to capture the MNP‐coated WBCs and elute CTCs through the outlet.^104^ B, A herringbone pattern is integrated on the main parallel channels to force cells near the top surface of the channels onto the bottom surface. Magnetic grains are self‐assembled on the bottom surface of the channels to generate a large magnetic field and field gradients. Cells larger than the underpass gap are captured, while smaller cells pass through the gap.^116^ C, A microfluidic device is composed of two isolate microfluidic devices that house three different microfluidic components: the DLD to remove nucleated cells from whole blood by size‐based deflection, inertial focusing to align cells to prepare for precise magnetic isolation, and magnetophoresis for sensitive isolation of bead‐labeled WBCs and unlabeled CTCs.^117^ D, The integrated device includes the isolating chip and the purifying device: the DLD structure with a tilted angle of 3.2° toward the fluid flow direction; the purifying device consisting of a purifying chip; and the permanent magnet and the stepping motor^118^

An integrated microfluidic chip called the μ‐MixMACS chip^104^ includes a microfluidic multi‐vortex mixing module and a magnetically activated cell sorting module. The first module can effectively bind MNPs coated with the CD45 antibody and WBCs. The second module captures the WBC within the channel and minimizes the interference of WBCs during subsequent CTC analysis, as shown in Figure 11A) The two modules are tightly connected together by a continuous flow path. A hybrid magnetic/size sorting (HMSS) chip with an asymmetric herringbone structure^116^ enhances mixing and ultimately deplete WBC fractions, as shown in Figure 11B. The system first uses a self‐assembled magnet to generate high magnetic forces that can eventually remove abundant WBCs. A single CTC was captured unbiased at a predetermined location when the sample passes through the size‐sorter region with a cutoff of 5 μm.

Another negative selection technique is to place a deterministic lateral displacement (DLD) within a microfluidic chip (Figure 11C) that continuously isolates WBCs and CTCs from whole blood based on their size differences. The inertial focusing induced by the DLD structure can precisely position these cells, while the magnetic isolation of the CTCs occurs in the microfluidic magnetophoretic region.^117^ To prevent clogging, blood must be diluted before it is added to the microchannel of the DLD structure. Some WBCs may be stuck in the corners of the herringbone structure. Accordingly, Jiang et al^118^ improved the DLD structure with a tilted angle of 3.2° toward the fluid flow direction and developed an automatic magnetic purifying device for negative isolation to enhance the purity of CTCs, as shown in Figure 11D.

Over the past decade, various CTC isolation methods using microfluidic systems have been analyzed using the positive magnetophoresis mechanism. CTC isolation in a microfluidic system, including the (a) efficiency of isolation, (b) sensitivity of detection, (c) accuracy of analysis, and (d) viability and purity of CTCs, has greatly improved by adjusting the nature of MNPs and the structure of microfluidic systems. The combination of several microfluidic systems not only enables multidimensional manipulation of CTCs but integrates isolation, detection, and analysis in a microfluidic chip.

However, this approach that uses antibodies against a surface antigen such as EpCAM to select cells of epithelial origin has potential limitations. (a) CTCs from non‐epithelial cancer will be missed in EpCAM‐based enrichment; (b) CTCs may not be fully captured due to the weakening or loss of EpCAM expression caused by EMT; (c) MNPs coupled to the surface of the CTCs may be phagocytosed by the cells to induce toxicity, which alters the original state of cells and reduces the reliability of biological studies. Moreover, although negative selection using antibody mixtures for cancer type‐specific antigens can expand the cell capture of the CTC antigen‐dependent capture method, issues still need to be determined, such as (a) whether WBCs can all be labeled owing to their large number and (b) whether CTC can be damaged by large mechanical stress owing to the existence of an HB or a DLD structure.

Therefore, breaking the limitations of existing technologies and developing an integrated next‐generation CTCs isolation system will have far‐reaching implications for cancer diagnosis and treatment. The platform is capable of (a) effectively isolating individual cells and clusters simultaneously, (b) providing a pure cell population with minimal or no contamination of normal blood cells, and (c) high‐throughput recovery of cells with greater viability. Moreover, the system can be easily applied to multiple positive or negative selection methods for diverse cancer cell lines with various surface markers and eventually become a single‐use tool available in clinical testing.

## 4 LABEL‐FREE ISOLATION OF CTCS

The key to the method of magnetically isolating labeled CTCs is the selection of biochemical markers for epithelial cells, but the expression of epithelial markers is reduced or even eliminated after CTCs undergoes EMT, which is a significant defect of this method. The technology developed in recent years for label‐free magnetic isolation of CTCs can potentially solve this problem. This approach may provide more sensitive CTCs isolation as well as more analytical alternatives and offer more accurate disease assessments to personalize treatment and evaluate drug efficacy.^23^

The main principles of label‐free isolation are based on the physical properties of the cell, such as differences in cell size and shape, and the difference in magnetic susceptibility of the medium in which the cells are located. On the one hand, since most CTCs are larger than normal blood cells, they will be subjected to a greater magnetic force in the presence of a magnetic field. On the other hand, the magnitude of the magnetic force can be controlled by the magnetic susceptibility of the medium. All biological particles (including CTC) except WBC exhibit diamagnetism. Only a few chemicals, such as bismuth (III) subsalicylate, have better diamagnetism than water, but they dissolve under strong acid conditions to result in poor biocompatibility.^119^ In contrast, it is reasonable to choose a magnetic liquid to change the magnetic susceptibility. Two types of magnetic liquids have been used to isolate diamagnetic particles: paramagnetic salt solutions and ferrofluids. Both isolate various biological cells without largely affecting the cell viability.

### 4.1 Types of medium

Ferrofluids and paramagnetic salt solutions are magnetic liquids that are often used to isolate particles with nonmagnetic properties, including most synthetic or biological particles. Magnetic labeling entails a considerable amount of time to perform chemical reactions and requires the removal of surface marker to obtain a pure sample after the isolation. In contrast, the method based on negative magnetophoresis can significantly facilitate the preparation and post‐analysis of samples, and further greatly improve the efficiency and purity of cell isolation.

#### 4.1.1 Paramagnetic salt solutions

Paramagnetic salt solutions, such as manganese (II) chloride (MnCl_2_) and gadolinium (III) chloride (GdCl_3_) solutions, are transparent, which facilitate the observation and recording of the particle/cell isolation process. Owing to the relatively low magnetic susceptibility of paramagnetic salt solutions, the salt concentration must remain high to generate sufficient magnetophoresis. This requirement results in a significant increase in the density of the paramagnetic solution such that the particles/cells rise rapidly in solution and are captured on the upper surface of the microchannel. Therefore, the effective isolation of particles/cells in the paramagnetic salt solution can be improved by reducing the distance of permanent magnets to the microchannel or increasing the magnetic field gradient by combining with the soft magnets.

Particles and cells are continuously controlled in a paramagnetic solution using two closely facing magnets around the capillary.^120^, ^121^ The distance between the permanent magnet and the microchannel can be reduced to within 300 μm by embedding the permanent magnet into the polydimethylsiloxane (PDMS) chip.^122^ This process is capable of continuously isolating nonmagnetic particles in a paramagnetic solution. A microfabricated ferromagnetic (Nickel) structure may also be embedded into a microfluidic chip to provide a strong magnetic field gradient because the structure can concentrate magnetic flux lines from external permanent magnets.^119^

Although paramagnetic salt solutions can be used in label‐free isolation, they exhibit excellent performance in static flow applications, such as density measurement. The reason is that paramagnetic solutions containing transition metals and lanthanide metals have weak magnetic properties owing to the magnetic moments generated by their unpaired inner shell electrons. By contrast, owing to their enhanced magnetic properties, ferrofluids are more suitable for applications such as high‐flux isolation requiring a continuous flow.

#### 4.1.2 Ferrofluids

Ferrofluids have higher volume magnetic susceptibility and magnetization under a magnetic field generated by the permanent magnet. A ferrofluid stream having a predefined concentration promotes negative magnetophoresis and can isolate CTCs from the particle/cell mixture. Ferrofluids are stable colloidal suspensions containing a single magnetic domain with a diameter of about 10 nm. MNPs usually consist of Fe_3_O_4_, which is stably dispersed in water or oil by coating a layer of surfactant. As opaque liquids, ferrofluids require fluorescent staining to observe suspended particles.

Commonly used ferrofluids for particle/cell isolation include commercial EMG 408 or 707. Ferrofluids can also be synthesized using appropriate salt concentration, tonicity, and surfactant to improve biocompatibility; these are referred to as customized ferrofluids. Diamagnetic particles in the EMG 408 ferrofluid are focused by flowing through a T‐microchannel with a single permanent magnet,^123^ which are valuable in subsequent isolation operations. A hybrid microfluidic technique based on the EMG 408 ferrofluid combines passive inertial focusing with active magnetic deflection to isolate diamagnetic particles by size.^124^ The diamagnetic particles are introduced into a circular chamber to investigate the extent of their deflection under the action of a nonuniform magnetic field.^125^ Two‐stream and three‐stream ferrofluid configurations are evaluated to determine the optimal isolation performance.^126^ Both configurations use the EMG 707 ferrofluid as the medium. In addition, a microfluidic device was fabricated via ultraviolet lithography to isolate diamagnetic fluorescent carboxy microparticles (∼4.5 μm) in pH 7 ferrofluids composed of magnetite nanoparticles.^127^ The pH 7 ferrofluid is the ideal magnetic fluid to use for the isolation of biological particles. More applications of label‐free isolation technology based on EMG 408 or customized ferrofluids are listed in Tables 3 and 4, respectively.

**Table 3:** Summary of sheathless flow microfluidic systems with one inlet

System	Diamagnetic particles (Diameter‐μm)	Media (ferrofluids)	*Q*	*η* (%)	Clinical validation	References
T‐shape	2.85 (magnetic) & 10	0.1 × EMG 408	240 μL/h	~100	No	[128]
	2.85 (magnetic) & 9.9	0.05 × EMG 408	55 μL/h	~100	No	[129]
U‐shape	5 & 15	0.01 × EMG 408	0.7 mm/s	~100	No	[130]
	5 & 15	0.5 × EMG 408	460 μL/h	~100	No	[131]
Groove	6 (magnetic) & 13	0.05 × EMG 408	30 μL/min	>95	No	[132]
Magnet	yeast cells/3 & 10	0.05 × EMG 408	0.5 mm/s	~100	No	[133]
	3.1 & 4.8	0.5 × EMG 707	10 μL/min	—	No	[134]
	5	0.2 × EMG 408	120 μL/h	~100	No	[135]

**Table 4:** Summary of sheath flow microfluidic systems with two/three inlets

System	Diamagnetic particles (Diameter‐μm)	Media (ferrofluids)	*Q*_s_ & *Q*_p_	*η* (%)	Clinical validation	References
Ferrofluid sheath	1/1.9/3.1 & 9.9	Diluted EMG 408	3 & 10 μL/min	~100	No	[136]
	CCL‐2 & 5.8/RBCs	Customized	8 µL/min	>99	No	[137]
	H1299/A549/H3122/PC3/MCF7/HCC1806 & WBCs	Customized	6 & 6 mL/h	92.9	Yes	[138]
	D‐5.1/L‐7.7 & 6	0.3 × EMG 408	6 & 120 μL/h	~100	No	[139]
	4.5 & 5.5 & 6.2 & 8.0 ‐yeast cells	0.1 × EMG 408	9 & 180 μL/h	‐	No	[140]
Water/buffer sheath	10 & 20	0.75 × EMG 408	3 & 1 mL/h	~100	No	[141]
	A549/H1299/MCF‐7/MDA‐MB‐231/PC‐3 & WBCs	Customized	1.2 mL/h	82.2	No	[51]
Magnet	E. coli cells & 7.3/S. cerevisiae cells & 1	EMG 408	6 & 1.5 μL/min	~100	No	[142]
	8 & 10/U937 & RBCs	Gd‐DTPA	0.32 μL/min	>90	No	[119]
	2 & 7	0.5 × EMG 408	3 μL/min	—	No	[143]

### 4.2 Types of microfluidic systems

#### 4.2.1 Simple microfluidic systems

Microfluidic technology has numerous advantages as a representative of a lab‐on‐a chip technology, including high throughput, integration, low cost, and small size. Microfluidic systems can be classified by the number of inlets in a microfluidic chip, as follows: a sheathless flow system (one inlet) and a sheath flow system (two/three inlets, one of which is the sheath flow). The sheathless flow system, distinguished based on the shape of the microchannel and the number of the magnet, is divided into subtypes: T‐shape, U‐shape, groove, and magnet. Meanwhile, the sheath flow system, classified according to the medium of sheath flow and number of magnets, is further divided into the following subtypes: ferrofluid sheath flow, water/buffer sheath flow, and magnet. Tables 3 and 4 list the types of particles/cells and magnetic fluids, volume flow rate (*Q*), and isolation efficiency (*η*) in various simple microfluidic systems.

**Figure 12:**
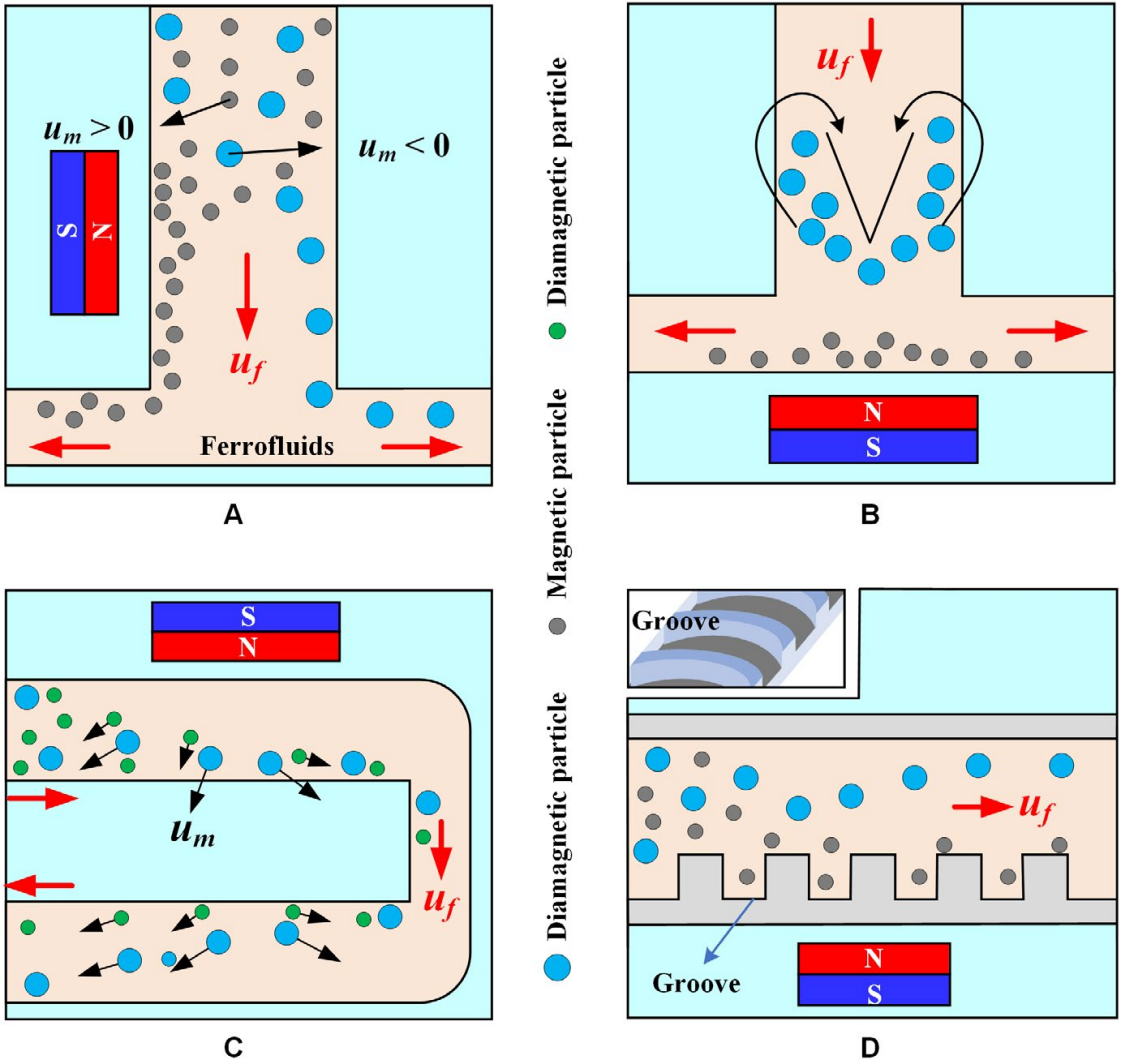
Sheathless microfluidic system with different microchannels. Schematic of the isolation of magnetic particles from diamagnetic particles when a permanent magnet is placed (A) one side^128^ or (B) at the top^129^ of a T‐shaped channel. C, Mechanisms of sheathless size‐based magnetic isolation of diamagnetic particles in a ferrofluid.^130^ D, Structure of the groove and spatial distributions of particles. Magnetic particles migrate to the centerline of the channel, whereas diamagnetic particles are focused onto the sidewalls^132^

Figure 12 describes the existing strategies of particle isolation in a microfluidic system with sheathless configuration, in which T‐shaped, U‐shaped, and grooved channels were adopted. The throughput of magnetic and diamagnetic particle isolation in a T‐shaped microchannel can be significantly improved by replacing the diamagnetic aqueous medium with a dilute ferrofluid, as shown in Figure 12A. In water‐based isolation, the maximum flow rate of magnetic particles and diamagnetic particles is completely isolated at only 150 μL/h, while the isolation in diluted ferrofluids reaches 240 μL/h, which reflects a 60% increase in throughput.^128^ A single permanent magnet was placed on top of the T‐shaped microchannel to continuously capture and pre‐concentrate the diamagnetic particles in the ferrofluid stream (Figure 12B), allowing both magnetic and diamagnetic particles to be simultaneously captured at different locations in the microchannel.^129^ Alternately, a single permanent magnet was placed over the entrance of the U‐shaped microchannel (Figure 12C), the particles are magnetically focused at the inlet, and then continuously separated into two streams in the outlet by size‐dependent magnetophoresis.^130^ The results show that increasing the outlet width of the U‐shaped channel can significantly enhance the diamagnetic particle isolation in ferrofluids.^131^ Moreover, a microfluidic device that couples microvortex and magnetophoresis was developed to isolate magnetic and diamagnetic particles with high throughput.^132^ This device exploits positive magnetophoresis and microvortices generated by grooves to focus magnetic particles near the centerline of the channel, while diamagnetic particles are focused on the side wall of the channel under the action of negative magnetophoresis and hydrophoresis, as shown in Figure 12D.

**Figure 13:**
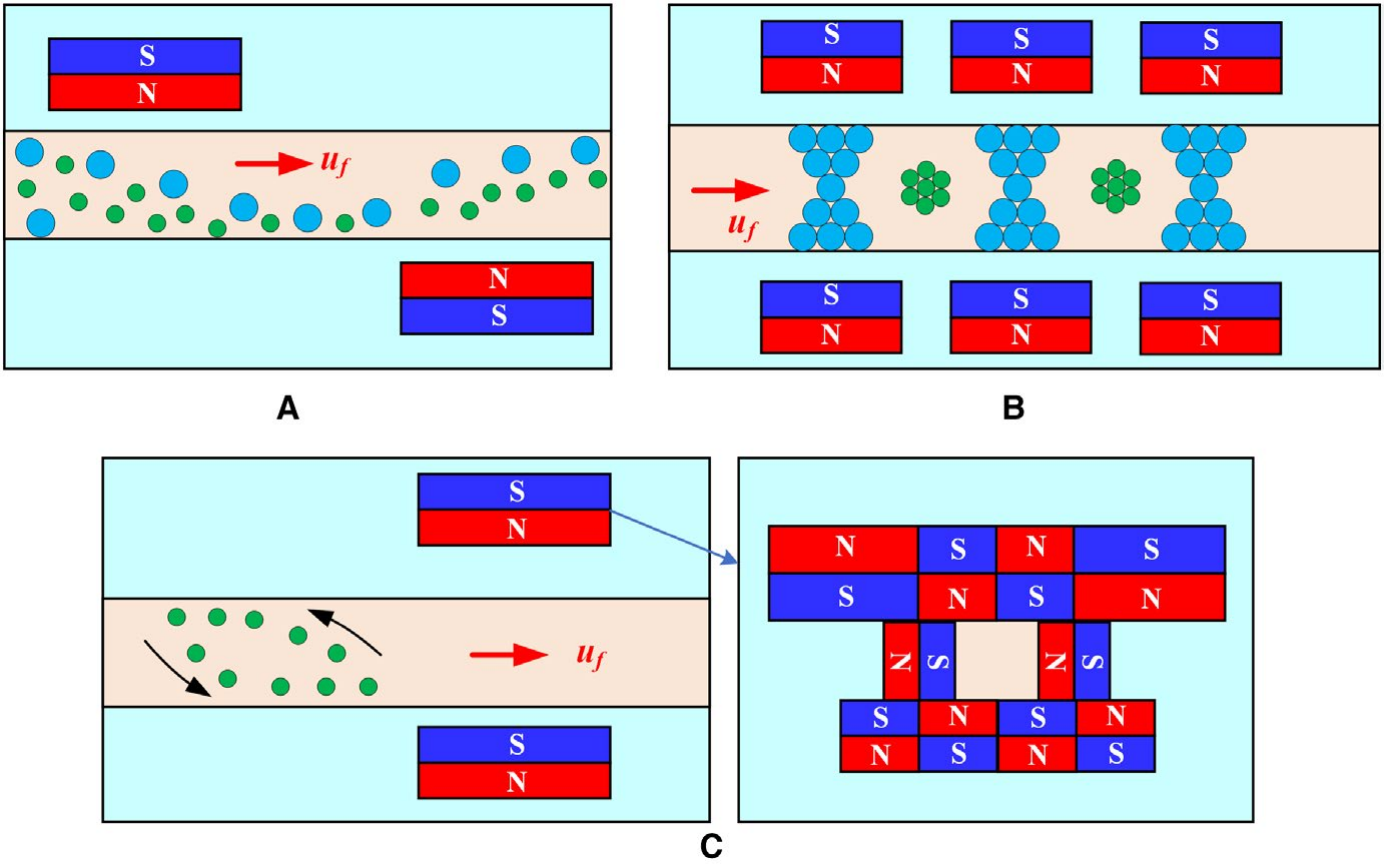
Sheathless microfluidic system with multiple magnets. A, Mechanism of diamagnetic particle and cell isolation in a ferrofluid flow through a straight microchannel using two offset magnets.^133^ B, Schematic of an array with two magnets symmetrically placed to isolate diamagnetic particles/cells of different sizes.^134^ C, Asymmetric magnet configuration to embed the two attracting permanent magnets into PDMS and form a single asymmetric cycle of concentrated particles in the microchannel^135^

The number and position of the magnets directly affect the movement of particles/cells in a straight channel, as shown in Figure 13. Two permanent magnets are spatially staggered on both sides of the straight microchannel, and the distance between the permanent magnet and the microchannel is different (Figure 13A). The purpose of the first magnet is to focus the particle mixture into a single stream, and the second magnet is intended to deflect particles of different sizes into the associated flow paths for continuous isolation.^133^ An array of magnets in which two permanent magnets are symmetrically placed along both sides of a straight channel can create multiple isolation zones with minimum magnetic field strength along the centerline of the channel to isolate diamagnetic particles of varying sizes,^134^ as shown in Figure 13B. A microfluidic system with an asymmetric magnet configuration can form a single asymmetric cycle of concentrated particles in the microchannel (Figure 13C) and maintain its size and position unless the flow of ferrofluids is increased.^135^

**Figure 14:**
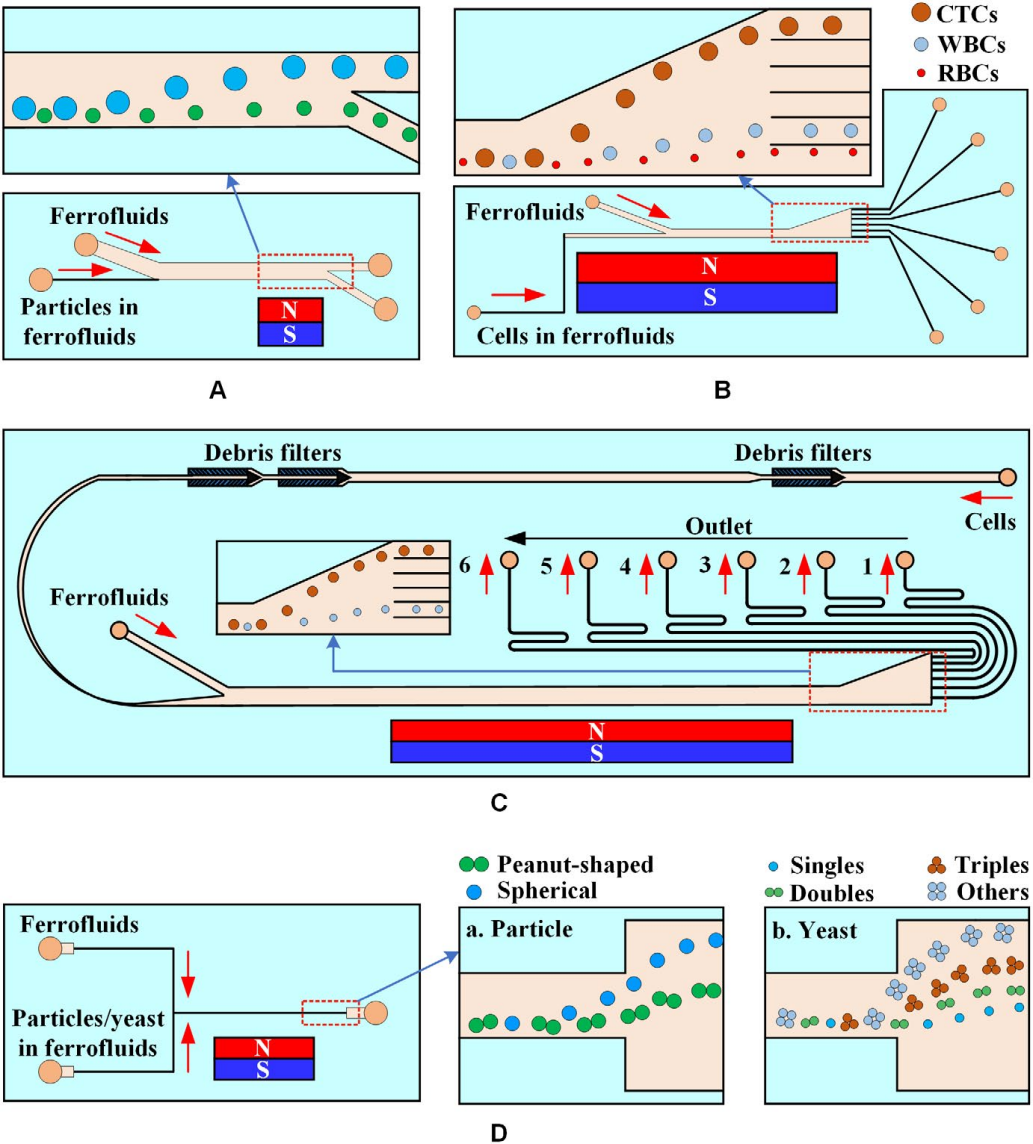
Ferrofluid sheath microfluidic system. A, Diamagnetic particles and a ferrofluid mixture are introduced into the microfluidic channel and hydrodynamically focused by a ferrofluid sheath flow from the other inlet.^136^ B, Mechanisms of spatial separation of cell mixtures at the end of the channel. Larger HeLa cells are deflected from their laminar flow paths toward the upper outlets by magnetic buoyancy forces.^137^ C, Cells in blood were first filtered to remove debris and then focused by a ferrofluid sheath flow. After entering the channel region on top of a permanent magnet, large cells, including CTCs and some WBCs, received a more size‐dependent magnetic buoyance force than those received by smaller WBCs. This process resulted in a spatial separation between them at the outlets.^138^ D, Shape‐based isolation schematic of (a) equal volume spherical and peanut‐shaped diamagnetic particle^139^ and (b) single, double, triple, and other yeast cells^140^

Shear flow isolation technique first focuses diamagnetic particles/cells to one side of the microchannel using inherent lift or resistance caused by fluid and channel structures and then isolates them based on their size or shape under a magnetic field. Ferrofluids, water, or buffer can be used as a source of sheath flow. Zhu et al^136^ used the ferrofluid sheath flow to continuously isolate three sets of diamagnetic particles of different sizes under a static magnetic field, as shown in Figure 14A. Zhao et al^137^ developed a biocompatible ferrofluid that not only maintains its colloidal stability under strong magnetic fields but maintains cell activity for up to 2 hours. Customized ferrofluids were used to isolate CTCs in a microfluidic system with six outlet channels, as shown in Figure 14B. They^138^ subsequently reported a ferrohydrodynamic cell isolation device containing three debris filters (Figure 14C) that can isolate various low‐concentration cancer cell lines from RBC‐lysed blood. Meanwhile, shape is an essential property of particles/cells that can provide useful information for cell synchronization or disease diagnostics. The equal volume spherical and peanut‐shaped diamagnetic particle mixture is prefocused to a tight stream by a sheath ferrofluid, which is then split into two substreams because of the shape‐dependent cross‐stream magnetophoretic motion,^139^ as shown in Figure 14D. Chen et al^140^ presented the continuous‐flow morphology‐based fractionation of a heterogeneous mixture of yeast cells treated in dilute ferrofluids. The isolation performance of this technique was evaluated by comparing the existing positions of four groups of yeast cells classified as singles, doubles, triples, and others, as shown in Figure 14D.

**Figure 15:**
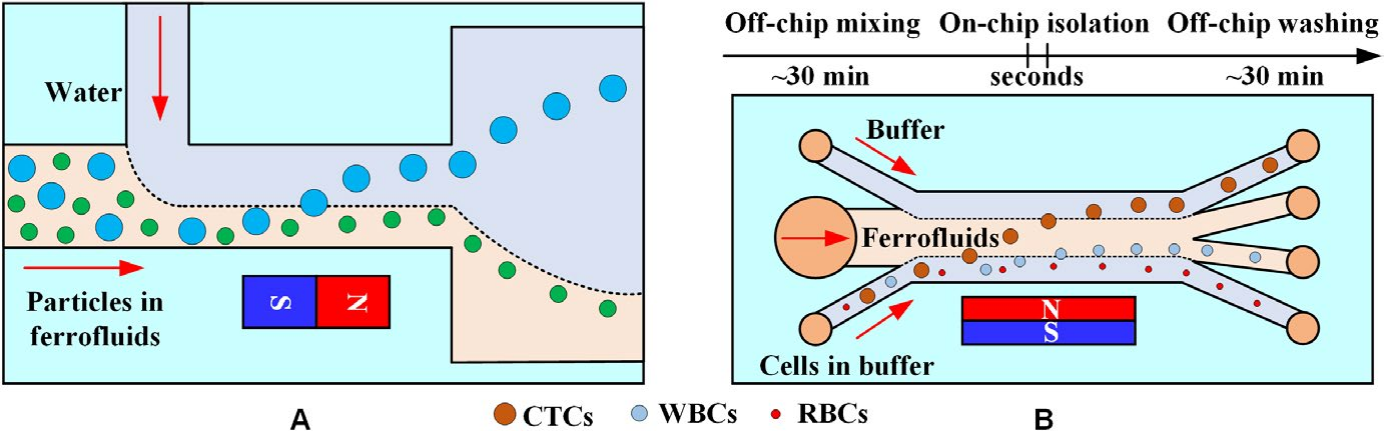
Water/buffer sheath microfluidic system. A, Mechanism of diamagnetic particle isolation and washing in an inertial ferrofluid/water co‐flow. Larger particles resuspend into the water across the ferrofluid‐water interface.^141^ B, Schematic of cell isolation in customized ferrofluids. The cell sample, ferrofluid, and buffer are injected into the device without premixing. CTCs are only in contact with ferrofluids when they are isolated from each other. CTC exposure to the ferrofluids is shortened to seconds^51^

The normal functions of a cell need to be maintained during and after isolation for post‐isolation analysis. Although ferrofluids exhibit a certain degree of biocompatibility, they are not the natural medium for cells, preventing the cells from remaining in the ferrofluid for a prolonged period. Therefore, an appropriate choice is to use water or buffer as the sheath flow. A high‐throughput microfluidic system has been developed to simultaneously separate and wash diamagnetic particles in a ferrofluid/water co‐flow.^141^ This method only transfers larger particles across the ferrofluid‐water interface, allowing them to resuspend into the water, as shown in Figure 15A. Zhao et al^51^ designed a three‐inlet microfluidic device (Figure 15B) in which customized ferrofluids and cells enter the chip separately rather than being premixed, reducing the exposure time of live cells to ferrofluids from hours to seconds and skipping the washing step as larger CTCs are resuspended into the buffer stream after isolation.

**Figure 16:**
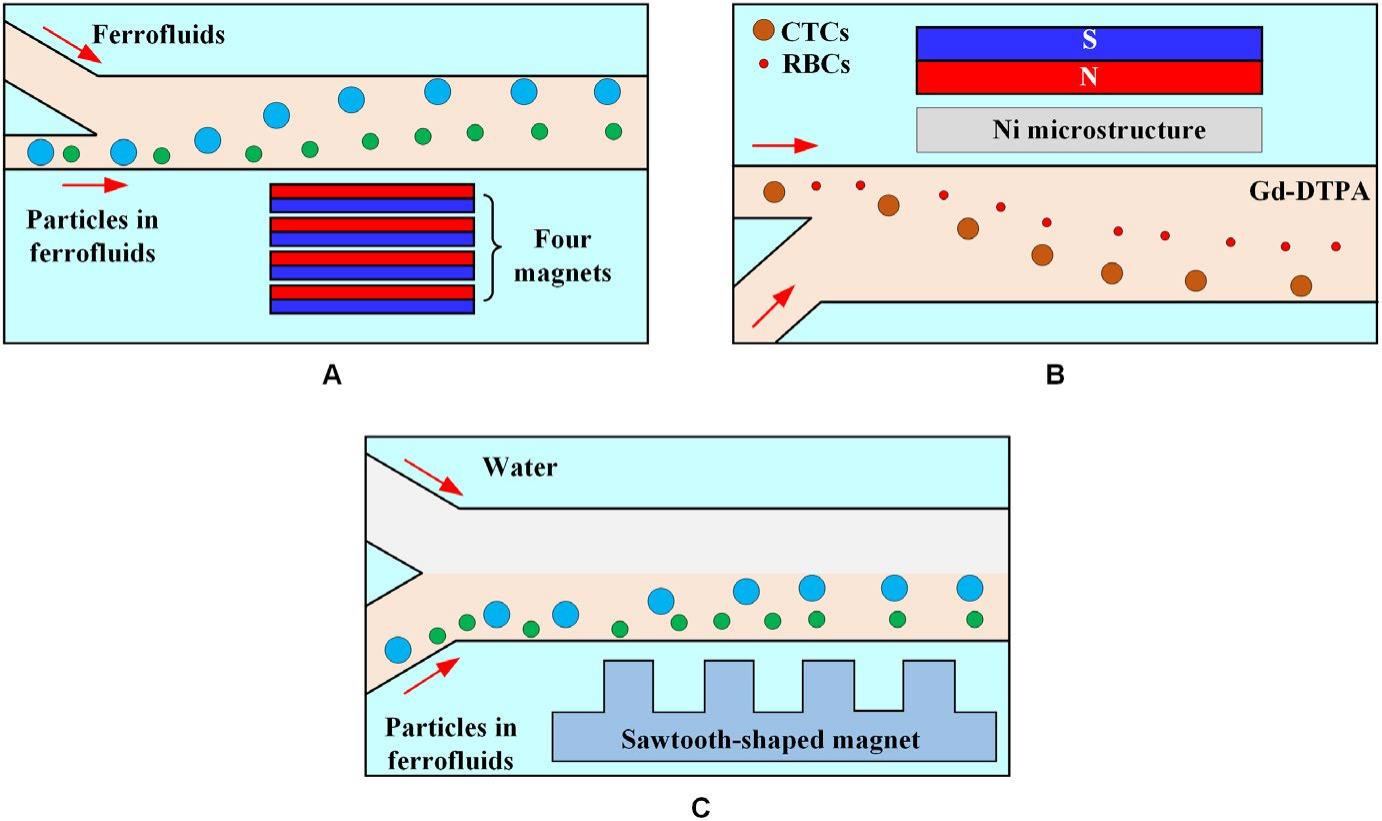
Sheath microfluidic system with different magnet structures. A, A stack of four permanent magnets was embedded into a PDMS chip with their magnetization direction vertical to the channel during curing.^142^ B, The particle flow is aligned with the wall near a nickel microstructure, where the maximum magnetic field gradient is achieved. When a magnetic field perpendicular to the direction of flow is applied by a permanent magnet adjacent to the nickel microstructure, cells in a paramagnetic solution are deflected laterally because of magnetic repulsion force.^119^ C, Mechanism underlying the isolation of particles with different sizes based on water sheath flow under a local magnetic force generated by microfabricated sawtooth‐shaped magnets^143^

Magnetic isolation of particles/cells depends on the magnetic field gradients and forces generated by the magnets. Zhu et al^142^ developed a microfluidic device with four permanent magnets to continuously isolate cells of different sizes on the basis of hydrodynamics, as shown in Figure 16A. Shen et al^119^ placed a nickel soft magnetic structure between the microchannel and the permanent magnet to enhance the local magnetic force, allowing the isolation of U937 cells from RBCs, as shown in Figure 16B. Zhou et al^143^ designed the magnet into a sawtooth shape to increase the magnetic force and used the laminar fluid interface of two co‐flowing fluids (ferrofluids‐water) to focus and isolate diamagnetic particles, as shown in Figure 16C.

#### 4.2.2 Integrated microfluidic system

Although both sheathless and sheath flow techniques can achieve particle/cell isolation, biocompatible ferrofluids or devices that resuspend cells into buffers have also been developed. These techniques only focus on the viability of the isolated cells and involve no further analysis of the cells, hence a integrated system is lacking. Possible reasons can be: (a) The strategy of integrating multiple isolation techniques to accurately separate cells is too complicated. (b) The existing magnetic isolation technology is immature, for example, ignoring cell deformability, which makes it more difficult to integrate other technologies. (c) High cost for integrating. In addition, most label‐free magnetic isolation techniques are based on cell size and may lose small CTCs that overlap in size with that of WBCs.

Therefore, although label‐free technology has emerged only recently, technical defects still arise, requiring innovation in its clinical application. Regarding labeled magnetic isolation technology, many microfluidic systems, including integrated systems, have been reported but have not been clinically applied. The development of a new generation of labeled technology to replace the first generation is expected to be significant for cancer treatment.

## 5 CONCLUSIONS AND OUTLOOK

Magnetic cell isolation plays a significant role in biology and medicine, where cells can be isolated under the combined force of biological bindings and magnetic fields. Given their significant prognostic or diagnostic value, CTCs that provide information on more specifically targeted treatments or contribute to the development of personalized medicine are particularly valuable. In this article, we review the methods of CTC isolation for positive magnetophoresis (labeled) and negative magnetophoresis (label‐free). We also discuss the mechanisms for these two magnetic isolation methods to facilitate understanding. Research on magnetically isolated CTC technology has progressed, particularly labeled CTC technology. For instance, CellSearch technology is the only technology approved by the United States Food and Drug Administration (FDA).

Some aspects of CTC magnetic isolation research are still unclear, especially the design of the channel structure inside the microfluidic system and the precise control of the external magnetic field. Many studies focus on simple descriptions of experimental phenomena rather than in‐depth theoretical analysis, failing to achieve accurate isolation of individual CTCs using magnetic fields. For positive magnetophoresis, finding reversible MNPs to obtain pure CTCs will be an important direction for future research. For negative magnetophoresis, eliminating the situation where particles rigidly replace living cells and shortening the exposure time of CTCs in magnetic fluids is an important direction for future research. Moreover, most of the existing techniques are carried out in the case of model validation instead of clinical validation with real patient blood. Therefore, the clinical validation of microfluidic system is urgent.

Furthermore, the magnetic isolation technology based on microfluidic system needs to break the limitations, such as low purity and efficiency. For CTC technology to progress, new techniques need to be developed to increase efficiency and thus provide a purer population with a larger number of viable cells available for additional downstream analysis. The ideal CTC isolation technology requires (a) versatility on capturing multiple heterogeneous cell populations, (b) ability to maintain cell activity in the chip channels, and (c) extensibility for cell analysis. Further cultivation and research can be carried out. In summary, the magnetic isolation of CTCs has already become an important field in microfluidic system research, but further efforts are required.

## CONFLICT OF INTEREST

None.

## AUTHOR CONTRIBUTION

Yongqing He proposed the concept and supervised. Laan Luo consolidated data from literature, designed the illustrations, and drafted the preliminary version of this manuscript. All the authors approved the final version.

## Data Availability

All data generated or analyzed during this study are included in this published article.
